# Associations between training behaviors and injuries in performance-oriented parkrunners using traditional statistical methods and machine learning

**DOI:** 10.1371/journal.pone.0350075

**Published:** 2026-08-19

**Authors:** Han Wu, Katherine Brooke-Wavell, Clare Stevinson, Hui Fang, Keyi Zhong, Richard C. Blagrove

**Affiliations:** 1 National Centre for Sport and Exercise Medicine, School of Sport, Exercise and Health Sciences, Loughborough University, Loughborough, United Kingdom; 2 Department of Computer Science, Loughborough University, Loughborough, United Kingdom; 3 Center for Information Management, School of Business and Economics, Loughborough University, Loughborough, United Kingdom; University College Dublin, IRELAND

## Abstract

**Background:**

parkrun is the most popular recreational running event in the UK, yet running-related injuries (RRIs) pose a threat to participation. Training behaviors are likely to influence the likelihood of RRIs with strength and conditioning (S&C) activities widely believed to reduce RRI risk. This study aimed to examine associations between S&C training habits plus broader training behaviors and RRIs using traditional statistical methods and machine learning (ML).

**Methods:**

parkrun participants completed an online survey including questions about personal characteristics, training behaviors, and RRIs during the past 12 months. Chi square and Mann-Whitney U tests were used to investigate associations between training behavior and RRIs. ML classifiers were employed to establish multi-factorial prediction of RRIs. Shapley Additive Explanations were used to explain how frequently selected features affect RRI probability.

**Results:**

1,203 responses were obtained and 570 performance-oriented parkrun participants were used for data analysis. Resistance training (Φ = 0.099, *p* = 0.018) and stretching/yoga (Φ = 0.095, *p* = 0.023) were positively associated with RRIs. No other S&C or running training behaviors were associated with RRIs. ML classifiers achieved moderate predictive capacities and found non-linear associations between RRIs and number of interval training sessions per week, a positive association with stretching/yoga, and a negative association with coach-guided training.

**Conclusion:**

Participation in S&C activities is not associated with a lower likelihood of RRIs in parkrunners and resistance training and stretching/yoga was linked to greater likelihood of RRIs. ML classifiers suggest there is benefit to performing a high frequency of interval training sessions, excluding stretching/yoga, and having qualified coaches prescribe training.

## Introduction

Endurance running is a popular sport associated with a myriad of cardiometabolic and psychological health benefits [1,2], however; the activity causes musculoskeletal injury in a high proportion of participants even at a recreational level [3]. Running-related injuries (RRIs) are associated with drop-out from running events [4] and can often carry a high financial burden [5]. Numerous risk factors have been identified for RRIs, including injury history, age, impact loading, running kinematics, training behaviors, and neuromuscular weakness [6]. However, evidence is inconsistent and the combined influence of multiple risk factors is rarely considered.

‘parkrun’ is a free, organized weekly 5 km running/walking event held on Saturday mornings with approximately 330,000 runners/walkers taking part each weekend [7], making it the most popular recreational running event in the UK. Regular participation in parkrun has been associated with improvements in physical and psychological health [8]. Despite the popularity and benefits, data suggests that around half of parkrun’s participants are carrying a current injury, and most (86%) take part despite experiencing pain affecting their performance and wider training practices [3]. Clearly, RRI represents a potential threat to parkrun participation and its associated benefits, and therefore understanding how exercise training behaviors may modify injury risk in this sub-population of runners is important.

A plethora of scientific research has explored the efficacy of strength and conditioning (S&C) based injury prevention strategies in recreational sports participants [9]. Results generally show a positive link between S&C interventions and reduced injury occurrence. In endurance runners specifically, the evidence is far less clear [10,11], with only supervised exercise programmes tending to show a benefit in terms of injury prevention [12]. Competitive endurance runners often use S&C activities with the belief they help prevent injury [13], and a high proportion of running coaches/leaders also advocate the use of S&C as a ‘prehabilitation’ tool [14]. Despite these anecdotes, there is currently a lack of data documenting the extent to which recreational runners engage with S&C related practices and whether these, along with other, training behaviors are associated with RRI.

Machine learning (ML) is an extension of traditional statistical approaches that provides a greater level of depth compared to traditional statistics. To-date, ML has shown considerable potential in prediction and prevention of injury [15] in team sport settings; however, most studies used internal validation methods which may not withstand external prospective validation trials, thus it is unclear whether the acclaimed prediction performance were due to chance and overfitting. Additionally, the application of ML to training and injury data in individual sports is scarce [15]. Three investigations have employed ML to predict RRIs using anthropometric, biomechanical, and training load data during running [16–18], with only 2 studies including running training volume in their model [16,18], and only 1 that used S&C-related metrics [18]. More evidence is needed to evaluate the capability of ML for predicting RRIs in recreational endurance runners using training-load data. This information would be highly valuable in supporting the development of interventions to prevent RRIs in the future.

The primary aim of this study was to investigate the associations between S&C plus other training behaviors and RRIs during the past year in performance-oriented parkrun participants. The secondary aim was to evaluate the performance of ML algorithms in predicting RRIs using participant characteristics and training-related parameters.

## Materials and methods

### Survey design, ethics, and participants

An online 4-part, 27-question survey (Onlinesurveys, Jisc, Bristol, UK) was designed and approved by the Loughborough University Ethics Sub-Committee (#2021-6494-5371) and the parkrun Research Board to circulate to the parkrun community. The survey participants were ≥18 years old and had participated in at least one parkrun event. Participants provided electronic written consent. The survey was designed by two academic researchers (RB and CS), experienced in survey-based research. A pilot version of the survey was completed by 8 parkrun participants of both sexes, varying ages (21–67 years) and performance levels. Based upon qualitative feedback, the survey underwent further minor modifications before being launched.

The full set of survey questions can be accessed in S1 File Section 1. Section 1 of the survey collected personal characteristics including age, running experience, performance status, and motivations. Participants described their exercise and training habits over the last year in Section 2. This included the typical weekly frequency and duration of runs, interval training sessions, and S&C-related activities. S&C activities were categorized as: resistance training, plyometrics, core stability, bodyweight exercises, and stretching or yoga. Participants were also asked whether they participated in any other forms of exercise or sport on most weeks over the previous year. Section 3 of the survey captured muscle, joint and bone related problems experienced by participants over the last year and included questions from the Oslo Sports Trauma Research Centre Overuse Injury Questionnaire [19]. Section 4 questions related to a separate aim of the survey, thus data are not reported in this manuscript.

The survey was open for responses between 24^th^ August 2022 and 26^th^ December 2022. The UK parkrun blog, parkrun newsletter, and UK parkrun social media pages were used to promote the survey.

### Data filtering and descriptive statistics

To filter for performance-oriented runners, those who chose “Recreational without a focus on finishing times” for the question on level of participation, those who ran less than 3 times per week, and those whose running time per week was less than the combined time of doing other sports were excluded. See S1 File Section 2 for a more detailed description of the data filtering process.

### Statistical analysis

Statistical analyses were conducted using SPSS (IBM, New York, US). Chi square tests were used to assess associations between participation in each type of S&C activity and the prevalence of lower limb injuries (knee, ankle/Achilles, calf/shin, hip/groin, foot/toes, and thigh regions) during the past 12 months. Phi coefficient (Φ), which varies from −1–1 and represents the strength of correlation for a 2*2 contingency table, was used to quantify effect sizes. To reduce bias associated with participants engaging in S&C because of previous injuries, a filtered sub-set of data was created removing those who answered “reduce risk of a previous injury returning” and/or “rehabilitation of an existing or ongoing injury” as the reason(s) they conduct S&C. The same Chi square tests and Phi coefficient calculations were conducted on this filtered dataset. Mann-Whitney U Test was used to assess whether there is a significant difference in the running volume between the injured and the uninjured groups, where running volume was quantified as average number of running sessions per week and number of minutes of running per week. Rank-biserial correlation (r) was calculated to represent the relative size of the between-group difference. Chi square tests and phi coefficients were also used to assess the associations between participation in each type of S&C activity and whether the participant missed any parkrun events due to muscle/joint/bone problems during the past 12 months.

### Applying machine learning algorithms

Data preparation and application of ML algorithms were conducted in Python (The PSF, Delaware, US). Due to the unknown nature of the potential interactions among injury-inducing factors, several common feature selection methods and classifiers with different mathematical principles were tested for performance. Model performance was evaluated through the average area under the curve (AUC) of the Receiver Operating Characteristic (ROC) relationship during stratified 10-fold cross validation. See S1 File Section 3 for a workflow diagram.

#### Data preparation.

The outcome variable was identified as lower limb injuries experienced during the past 12 months (binary injured/uninjured). For the list of input features, please see S1 File Section 4. All binary variables were converted into 0/1 values, all continuous variables were normalized, and all nominal variables were one-hot encoded. When treating discrete variables (e.g., number of running sessions per week), one-hot encoding could reveal the effect of a particular training volume but may fail to capture continuous effects such as a linear relationship. As a result, two versions of the dataset were produced; one with discrete variables normalized and the other with them one-hot encoded. During feature selection, both datasets were tested and the better-performing one was selected for each classifier. When missing data referred to behavior (e.g., total minutes of running per week), it was assumed to be 0, otherwise (e.g., height/weight) it was assumed to be the median of all available samples.

#### Feature preprocess.

ML-related implementations were conducted using the sklearn module in python. Principal Component Analysis (PCA), Decision Tree Classifier, Relief, and Least Absolute Shrinkage and Selection Operator (LASSO) were used as feature/component selection methods (S1 File Section 5). Each method was tested on the following classifiers: Decision Tree, Random Forest, Support Vector Machine (SVM), K Nearest Neighbor (KNN), Naïve Bayes, Adaboost, Gradient Boosting, and Multilayer Perceptron (MLP) (S1 File Section 6). For PCA, the 27 components accounting for 90% of total energy were tested incrementally (i.e., test top 1 feature/component, then top 1 + 2, then top 1 + 2 + 3, etc.) on each classifier using stratified 10-fold cross-validation. The feature subset with the highest average AUC on the test sets for each classifier was returned for each feature selection method. For decision tree classifier and Relief as feature selection methods, features were ranked and then tested tested incrementally as in PCA components, and number of features tested was added 5 at a time until the best-performing feature subset for all classifiers fell within the range. For LASSO, α value was set to be equally spaced numbers within a designated range (e.g., take 30 equally spaced numbers between 0.01 and 0.0009 and test each number as α), and the features selected via each α were incrementally tested as per other feature selection methods. If the best-performing α value reached the boundary of the designated range, the range was widened until α fell within the boundary. The subset of features that achieved the highest average AUC on test sets for each classifier was taken forward to hyperparameter tuning.

#### Hyperparameter tuning.

Hyperparameter tuning was carried out by grid-searching a possible range of each hyperparameter on classifiers and retaining the combination with the highest average AUC using stratified 10-fold cross-validation. When a hyperparameter was numerical and the best-performing value was on the boundary of the set range, the range was widened and retested. When a hyperparameter fell within the boundary but lacked resolution, more numbers were added until the best-performing value remained unchanged (e.g., when 100 performs the best among [50, 100, 150], a further test was run with [75, 100, 125] until the best-performing value stabilized). For the best-performing hyperparameter combination, average AUC, average accuracy, average precision, and average f1 score across 10 folds were calculated. To compare the performance of ML classifiers with traditional statistical prediction, the statistically significant association with the largest effect size from previous calculations was also used to generate the above parameters.

#### Determining directions of associations.

Shapley Additive Explanations (SHAP) generated summary plots were used to determine the direction in which each feature selected by multiple classifiers predicts the outcome (S1 Section 7). Each classifier was trained again using the selected hyperparameters, and SHAP values for the positive class were calculated and used to produce a summary plot for every classifier. The plots were visually inspected to identify whether associations were positive, negative, or uncertain.

## Results

### Participant characteristics

A total of 1,203 full survey responses were collected, of which 570 remained after data filtering. Table 1 shows descriptive and injury-related statistics. Participants had 14.7 ± 13.9 years of running experience, and running duration per week was 3 h 47 min ± 2 h 45 min. Participants’ best 5 km performance was 26 min 46 s ± 5 min 54 s.

**Table 1:** General descriptive statistics and injury-related statistics.

Variable	Mean±SD (95% CI)
Age (years)	52 ± 12 (51, 53)
Sex (male, female, undisclosed)	293, 274, 3
BMI (kg.m^-2^)	24.2 ± 3.4 (23.9, 24.6)
Best 5 km performance during past 12 months	26min 46s ± 5 min 54s (26 min 17s, 27 min 15s)
Years of running/walking as a means for exercise	14.7 ± 13.9 (13.6, 15.9)
Duration of running per week	3h 47 min ± 2h 45 min (3h 33 min, 4h)
Participated in strength and conditioning activities during the past 12 months n (%)	371 (65.1)
Lower limb injuries n (%)	416 (73.0)
Number of days training was reduced due to lower limb injuries in the past 12 months	89.1 ± 137.2 (77.8, 100.4)
Forced to miss parkrun due to muscle, joint, or bone related problems during the past 12 months n (%)	362 (74.5)

Abbreviations: SD = Standard deviation. CI = Confidence interval. BMI = Body mass index.

### Strength and conditioning behaviors

**Fig 1:**
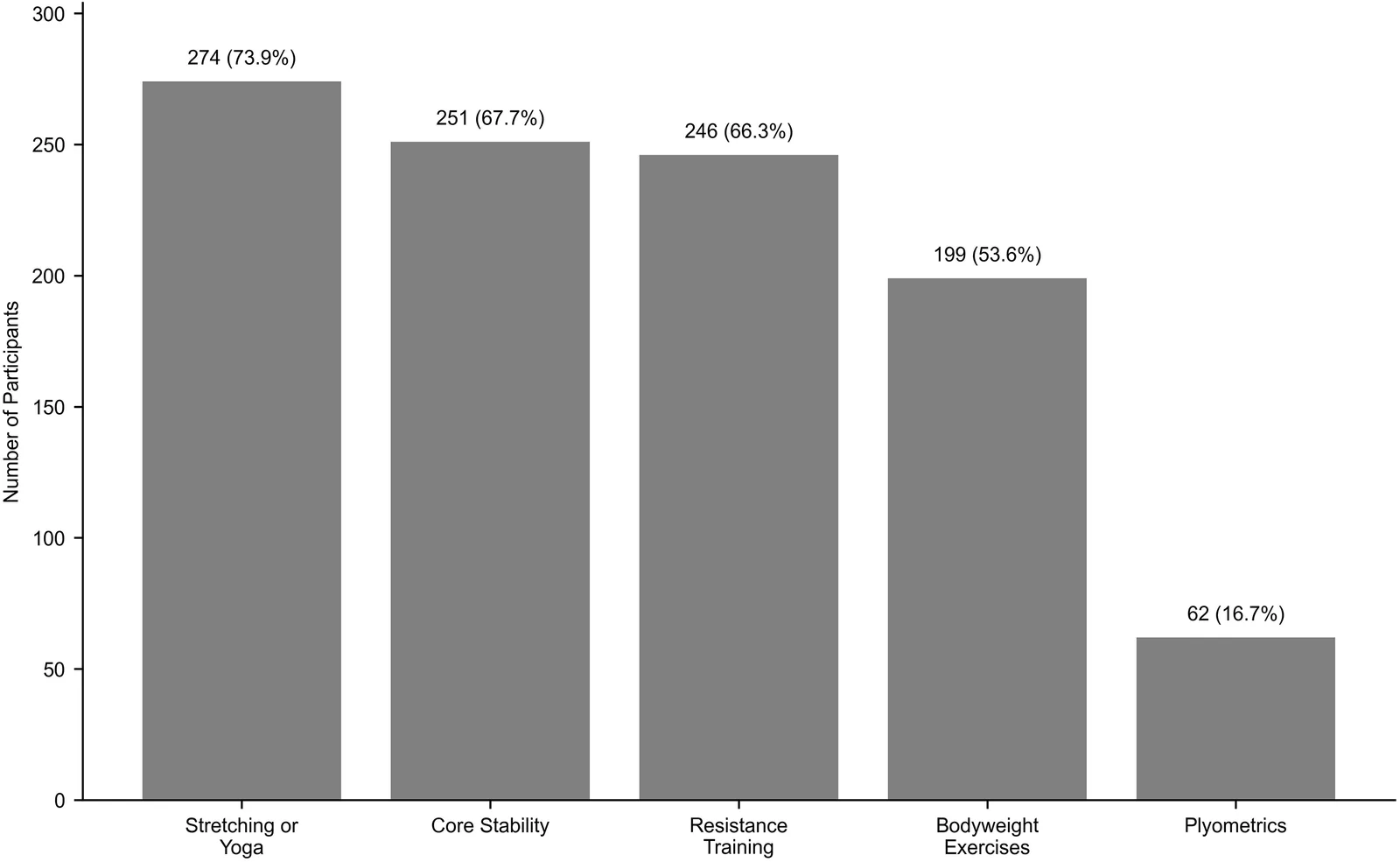
Number of participants performing each type of strength and conditioning activity on a regular basis during the past 12 months. Percentages refer to the proportions among participants who participated in S&C.

**Fig 2:**
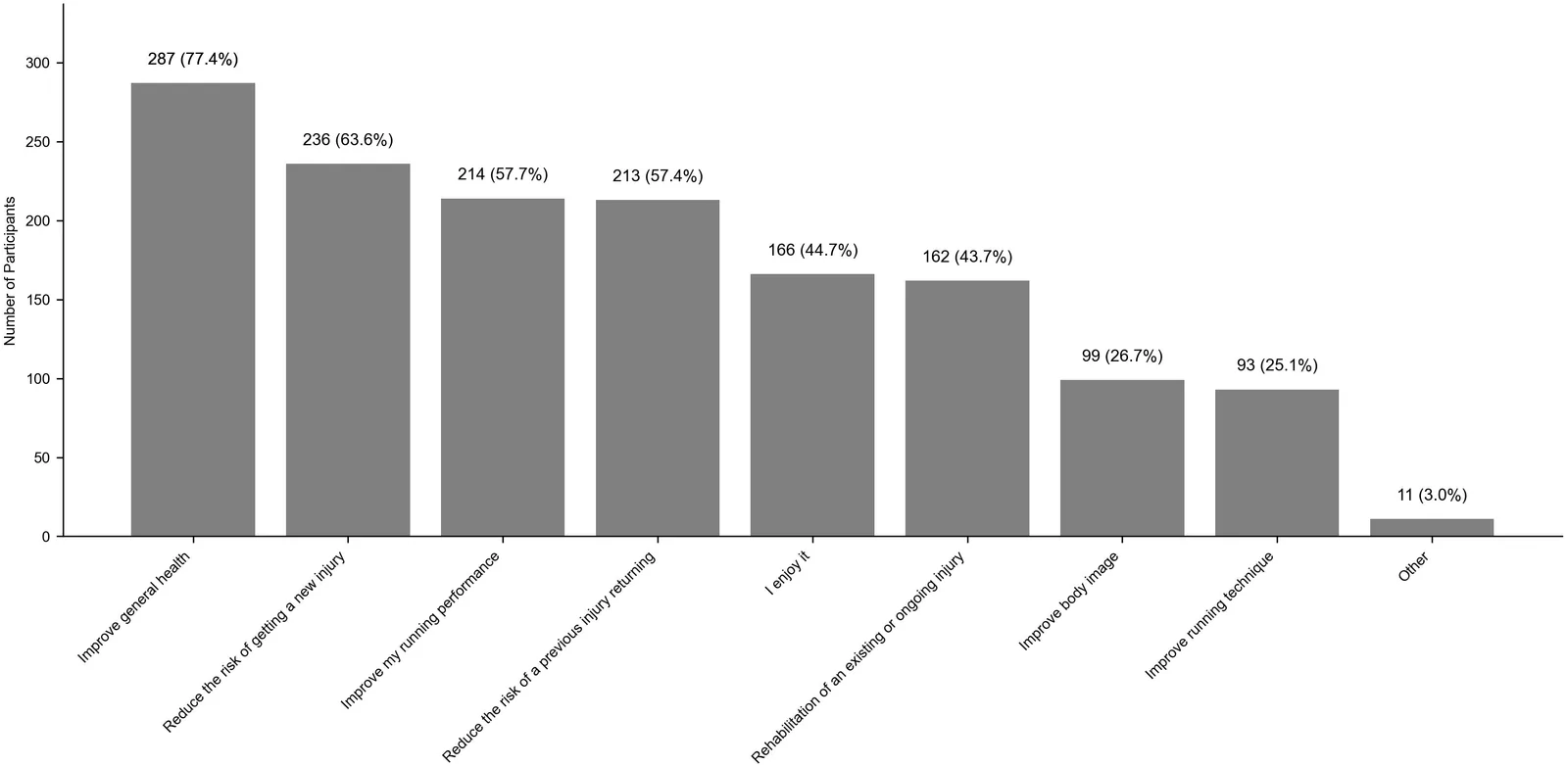
Reasons for including strength and conditioning activities. Percentages refer to the proportions among participants who participated in S&C.

Fig 1 shows participation in S&C activities among participants. Almost two-thirds (n = 371, 65.1%) of participants conducted some form of S&C regularly during the past 12 months, with the most popular modality stretching or yoga (73.9%), and the least popular plyometrics (16.7%). The most frequent reason for conducting S&C was to improve general health (77.4%), followed by to reduce the risk of getting a new injury (63.6%), to improve running performance (57.7%), and to reduce the risk of a previous injury returning (57.4%) (Fig 2).

### Physical problems/injuries

**Fig 3:**
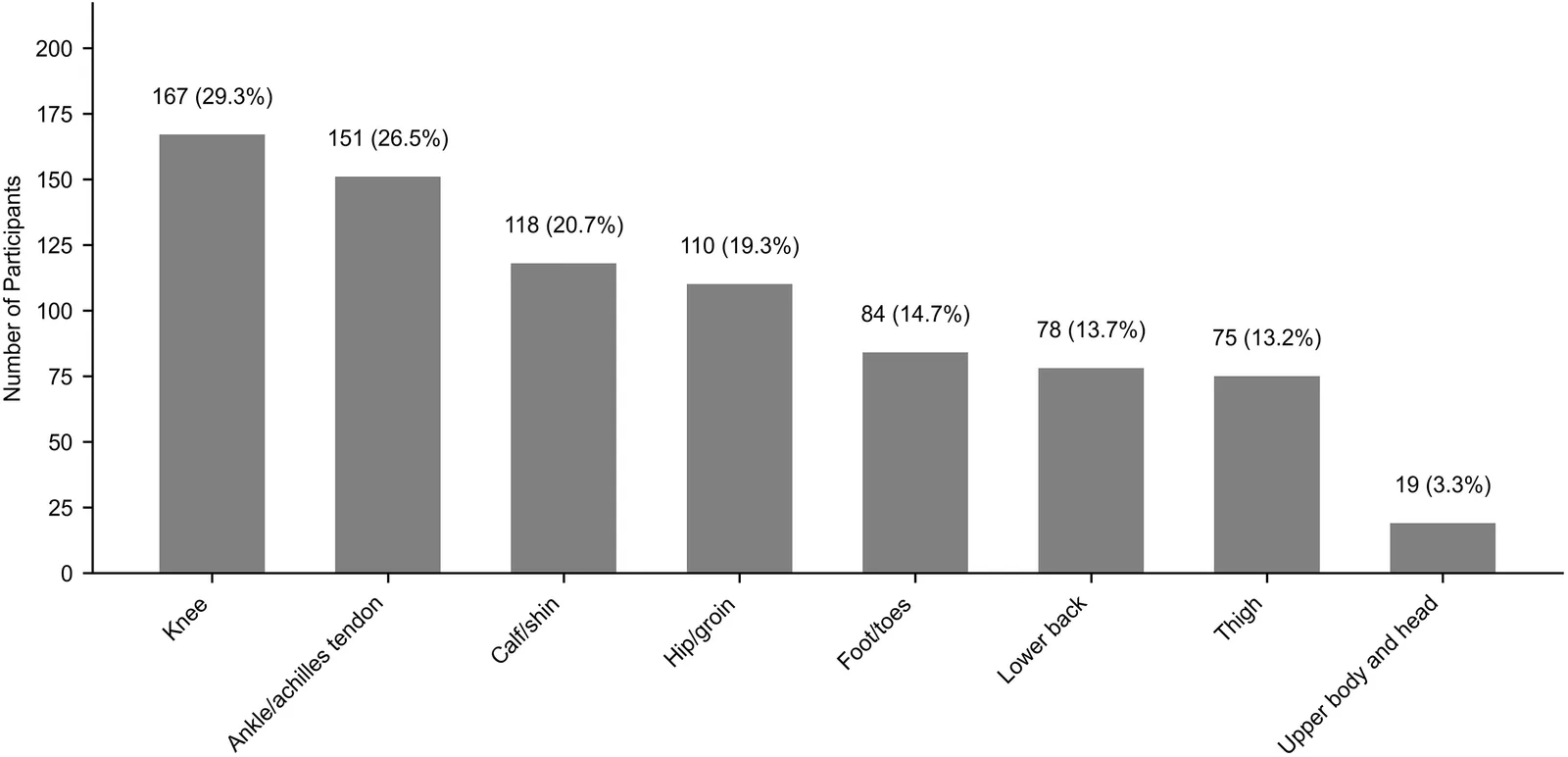
Number of participants who experienced injuries at different body locations during the past 12 months. Percentages refer to proportions among all participants.

Among the 570 participants, 73.0% had experienced at least 1 lower limb injury during the past 12 months, and 74.5% were forced to miss a parkrun event due to physical problems. The average number of days training was reduced due to lower limb injuries among all runners was 89.1 ± 137.2. Fig 3 shows the distribution of injury locations, with the knee (29.3%), ankle/Achilles tendon (26.4%), and calf/shin (20.7%) the most frequently injured sites.

### Association analysis

Table 2 shows the associations between S&C behavior or indices of running volume and lower limb injury prevalence during the past 12 months. When considering all participants, resistance training (Φ = 0.099, *p* = 0.018) and stretching/yoga (Φ = 0.095, *p* = 0.023) showed positive associations with lower limb injury prevalence. When only participants whose purpose for conducting S&C was not related to a previous injury were considered, no significant associations were found between each type of S&C modality and lower limb injury prevalence; however, stretching/yoga reached a level close to negative statistical significance (Φ = −0.109, *p* = 0.05). Frequency of running and duration of running per week showed no association with lower limb injury prevalence during the *p*ast 12 months. Table 3 shows the associations between S&C training behaviors and whether any parkrun event was missed due to muscle/joint/bone related problems during the past 12 months. As per the results above, resistance training (Φ = 0.145, *p* < 0.001) and stretching/yoga (Φ = 0.153, *p* < 0.001) were positively associated with missing a parkrun event due to *p*hysical *p*roblems.

**Table 2:** Associations between S&C training behavior or running volume metrics and lower limb injury prevalence during the past 12 months.

Exercise modality	Phi coefficient/Rank-biserial correlation	*p* value
Resistance training	0.099	0.018*
Plyometrics	−0.016	0.705
Core exercises	0.046	0.269
Bodyweight exercises	0.056	0.181
Stretching or yoga	0.095	0.023*
Frequency of runs per week	0.061	0.244
Duration of running per week	0.052	0.404
Subset of participants whose purpose of conducting S&C is not related to a previous injury (n = 321)		
Resistance training	0.004	0.946
Plyometrics	−0.044	0.429
Core exercises	−0.086	0.122
Bodyweight exercises	−0.028	0.613
Stretching or yoga	−0.109	0.050

*p < 0.05.

**Table 3:** Associations between S&C training behaviour and whether any parkrun event was missed due to muscle/joint/bone related problems in the past 12 months.

Exercise modality	Phi coefficient	*p* value
Resistance training	0.145	<0.001*
Plyometrics	0.031	0.463
Core exercises	0.026	0.529
Bodyweight exercises	0.074	0.079
Stretching or yoga	0.153	<0.001*

*p < 0.05.

### Application of machine learning algorithms

#### Feature preprocess.

Of the 4 feature/component selection methods, LASSO-ranked features yielded the best-performing feature subsets for all classifiers. Specifically, features ranked by LASSO and then fitted on each classifier incrementally and validated using stratified 10-fold cross-validation yielded the highest maximum AUC for all classifiers compared to the other feature selection methods. Among the 8 tested classifiers, Adaboost was the only method where the best model performance was achieved on the dataset treating discrete variables as normalized values. Model performances during feature selection stage are provided in S1 File Section 8, and features selected for each classifier are provided in S1 File Section 4.

#### Model performance.

Table 4 shows model performance after hyperparameter tuning for each classifier. ML classifiers achieved considerably better performance than the traditional statistical method in all parameters except precision, where the traditional statistical method (0.786 ± 0.077) outperforms all classifiers (0.731–0.763). Among the ML classifiers, average AUC ranged from 0.620 ± 0.058 to 0.694 ± 0.070. Multilayer perceptron (0.694 ± 0.070), gradient boosting (0.680 ± 0.075), and random forest (0.675 ± 0.076) achieved the top 3 average AUC among all classifiers. The top 3 average f1 scores were achieved by random forest (0.838 ± 0.028), gradient boosting (0.831 ± 0.026), and Naïve Bayes (0.830 ± 0.030). Random forest achieved the highest accuracy (0.733 ± 0.038) while multilayer perceptron achieved the highest precision (0.763 ± 0.029).

**Table 4:** Performance under the best-performing average (i.e., mean across 10 folds) AUC by each classifier.

	Average AUC ± SD	Average accuracy ±SD	Average precision ±SD	Average f1 score ±SD
Chi Square Association	0.557 ± 0.079	0.512 ± 0.060	**0.786 ± 0.077**	0.578 ± 0.061
Decision Tree	0.620 ± 0.058	0.697 ± 0.029	0.731 ± 0.014	0.816 ± 0.022
Random Forest	0.675 ± 0.076	**0.733 ± 0.038**	0.750 ± 0.019	**0.838 ± 0.028**
SVM	0.654 ± 0.065	0.696 ± 0.042	0.754 ± 0.024	0.806 ± 0.032
KNN	0.663 ± 0.051	0.698 ± 0.051	0.745 ± 0.025	0.810 ± 0.038
Naïve Bayes	0.658 ± 0.057	0.723 ± 0.049	0.754 ± 0.032	0.830 ± 0.030
Adaboost	0.632 ± 0.086	0.709 ± 0.047	0.750 ± 0.024	0.819 ± 0.030
Gradient Boosting	0.680 ± 0.075	0.723 ± 0.041	0.748 ± 0.029	0.831 ± 0.026
Multilayer Perceptron	**0.694 ± 0.070**	0.709 ± 0.052	0.763 ± 0.029	0.812 ± 0.040

Abbreviations: AUC = Area under the curve for the ROC curve.

#### Directions of associations.

Table 5 shows the directions of associations between features selected by multiple classifiers and the outcome. See S1 File Section 9 for SHAP summary plots for individual classifiers. Conducting 1 interval training session per week positively predicted lower limb injuries, while conducting 4 interval sessions each week predicted fewer injuries. Not conducting stretching/yoga negatively predicted lower limb injuries and conducting 3 sessions per week showed positive prediction, similar to the statistical association analysis finding. Following online training plans and not having a training plan positively predicted lower limb injuries, contrary to having a running coach creating the training plan.

**Table 5:** Directions of associations between features that appeared in more than 1 classifier and the outcome.

Feature name	Feature index	Direction of association in classifiers			
		Positive	Negative	Unsure	Total
Performing 1 interval training session per week	31	7	0	0	7
Performing 0 stretching/yoga sessions per week	70	0	7	0	7
Followed an online or app-based training plan, e.g., ‘couch to 5k’	80	6	0	1	7
Total number of parkruns completed	5	0	7	0	7
Best 5 km/parkrun performance during the past 12 months	6	0	6	0	6
Not having a training plan	81	5	0	1	6
Having a running coach plan for training	79	0	4	2	6
Performing 4 interval training sessions per week	34	0	4	1	5
Performing 0 interval training sessions per week	30	0	1	4	5
Performing 3 stretching/yoga sessions per week	73	3	1	1	5
Number of running sessions per week	23	0	5	0	5
Participation in sports other than running/S&C	15	4	0	0	4

Positive association means when the feature is positive/increases in number, injury becomes more likely.

## Discussion

This study aimed to investigate the associations between S&C training behaviors and injury prevalence during the past 12 months in performance-orientated parkrun participants. Based upon data from 570 respondents, participating in stretching/yoga and resistance training appears to increase the likelihood of an RRI, however these associations were no longer present when participants who conducted S&C because of a previous injury were removed. The study also aimed to examine the association between participant characteristics and training-related behaviors in predicting RRIs using ML. The ML algorithms achieved average AUC performances up to 0.694 ± 0.070, with random forest, gradient boosting, and multilayer perceptron the best performing classifiers. Among the selected features, 4 interval training sessions per week and completing no stretching/yoga were associated with decreased likelihood of injuries, while performing 1 interval training session and performing 3 stretching/yoga sessions per week were associated with higher injury occurrence. Participants who did not follow a training plan or followed an online training plan were more likely to report an injury; however, runners who followed a plan created by a running coach were injured less often.

Improving general health (77.4%) and improving performance (57.7%) were among the most popular reasons for conducting S&C. The use of resistance training to improve general health and to counter age-related physical problems has been consistently supported by research evidence [20], and there is good evidence that resistance training and plyometric-based exercises improve running economy and performance in long-distance runners [21]. Most participants (63.6%) engaged with S&C activities to reduce their risk of getting a new injury, which is a view shared by competitive runners [13], coaches, and running group leaders [14]. However, this rationale for S&C participation in runners is not well-supported by prospective training studies [10–12], nor the associations between S&C engagement and injury occurrence found within this study. Injury prevention as the basis for S&C provision in runners potentially originated from evidence that neuromuscular training can reduce risk of injuries in athletes participating in other sports [22]; however, the mechanisms of injury in these activities compared to endurance running are likely to be different. More well-controlled prospective investigations are required to validate the widely held belief that RRIs can be avoided with appropriate S&C prescription.

The reported lower limb injury prevalence was 73.0%, which was close to a previous study (75%) [23], but larger than another (55%) potentially due to less strict definition of injury [24]. Another study with a similar definition of injury also reported lower (54.8%) 1-year prevalence of injury, which shows that RRI prevalence vary across different cohorts [25]. Around three-quarters (74.5%) of participants also reported they had been forced to miss a parkrun in the last 12-months because of injury. The average number of days participants had reduced or missed training was cumulatively almost 3-months (89.1 ± 137.2 days), thus RRI represents an important potential barrier to participation for many performance-orientated parkrunners. Given the reported benefits of parkrun to health and wellbeing [8], advice related to injury prevention in parkrun participants should be the focus of future initiatives to reduce risk of drop-out.

Although a high proportion of participants believe that including S&C activities in their training program reduces their injury risk, participation in resistance training and stretching/yoga were found to be positively associated with lower limb injuries. This data supports observations from training intervention studies in runners [26], which concluded that stretching provides no protective effect against overuse injuries. It is interesting to note that within previous literature, prospective intervention trials either found no difference or a protective effect of S&C activities on RRIs [12], whereas a large-scale retrospective survey found that runners who stretch before running experienced more injuries, which is consistent with the current study [27]. Due to the retrospective nature of these studies, it may be possible that these positive associations between S&C engagement and RRIs are the result of participants becoming injured and commencing these activities as part of rehabilitation and/or reducing future injury risk. When only participants whose motivation for conducting S&C were not related to a previous injury were considered, effect sizes for associations decreased for all modalities, with stretching/yoga close to significance for a negative association (p = 0.05). Based upon these results it seems unlikely that S&C activities confers a reduced risk of suffering an RRI in performance-orientated parkrun participants. This aligns to findings from reviews of prospective training studies that showed no clear effect of participating in exercise-based prevention programs for preventing injuries in endurance runners [10,11]. No associations were found between running volume and lower limb injury prevalence. Conversely, 5 out of 8 ML classifiers identified number of running sessions per week as a feature that negatively predicts injuries, suggesting that an association may exist in a more complex form. Indeed, existing literature that has investigated training workload as a risk factor for RRI has shown conflicting results, which also points to potentially more complex relationships [28].

The performance of ML algorithms at predicting RRIs (mean AUC = 0.620–0.694) was better than a previous study (0.58–0.61) [16] and worse than two others (0.678–0.724; 0.70–0.74) [17,18]. The reason Saarela & Jauhiainen [17] achieved better model performance may be because they included biomechanical data on participants who were already injured. Differences in running biomechanics post-injury may be the result of the injury rather than the cause, which contributes to better model performance but reduces the model’s validity to predict injury. The investigation by Lovdal and colleagues [18] obtained daily training data prospectively and adopted a time-sequenced approach to construct features. The inherent high temporal resolution of the data may have contributed to better model performance. As the features used in the current study were almost entirely different to previous studies, it provides novel and valuable information on the ML predictions of RRIs.

Amongst the features selected for the best performing ML classifiers, performing 0 interval training sessions per week did not have a unidirectional relationship with injuries, performing 1 positively predicted injuries, while performing 4 negatively predicted injuries. This shows that the relationship between interval training session frequency and injury risk is not linear, and that performing a high number of interval training sessions per week may be beneficial. The complex nature of this relationship could explain the currently conflicting results surrounding the association between interval training sessions and RRIs [29,30]. The mechanism of such relationship is unclear but may involve other mediating factors such as a reduction in total running volume.

Another interesting finding is that participants who did not follow a training plan or who followed an online training plan were more likely to suffer RRIs. Conversely, runners who had their training prescribed by a running coach were less likely to get injured, highlighting the importance of working with a qualified coach. Previous evidence has shown that using self-devised training plans positively correlated with prevalence of RRIs [3], and that the use of running applications was not associated with altered RRI prevalence [31]. In addition, recent findings suggest that supervision by qualified professionals may be crucial for structured S&C programs to reduce the risk of RRIs [12]. Taken together, there appears to be benefits of runners working with qualified practitioners in the design and administration of training programs to reduce injury likelihood.

This study has several important limitations, which should be recognized. The main limitation of this study originates from its retrospective nature. It is not valid to infer causality from an observed correlation and thus no definitive conclusions can be drawn on the causes of RRIs. More importantly, the positive associations found between S&C training behavior and RRIs could be due to reverse causality (i.e., injuries leading to more S&C), as evidenced by its disappearance after excluding participants who conducted S&C to prevent or rehabilitate from an injury. A second limitation relates to sampling bias as the study title and aims may have attracted participants who were currently injured or had previously suffered from high numbers of RRIs. Another limitation is the lack of detail collected on training behaviors. A participant’s running volumes and intensities may vary considerably over 12 months but the survey question asked them to report typical training values. Similarly, it is likely that large inter-individual variability exists in the types of exercise and prescription under each S&C activity category. This could mask any beneficial effects of strength training particularly if evidence-based recommendations were not adhered to. Regarding the ML predictions of RRIs, a limitation exists in the lack of interpretability. To achieve predictions of RRIs with better clinical implications, it is recommended that new ML classifiers be developed that would enable a certain extent of prior knowledge input, leading to better interpretability and better alignment with known injury mechanisms, while still capturing the intricacies of the interactions among different features.

## Conclusion

Most parkrun participants with a performance-oriented goal engage with S&C exercises, with the belief they will reduce injury risk. Positive associations were found between RRIs and conducting stretching/yoga and resistance training; however, this is likely due to runners beginning S&C as a consequence of a previous injury. Thus, we conclude that S&C activities did not offer any protection against injury in this cohort of runners. ML algorithms achieved moderate level performance using features mostly pertaining to training behavior to predict RRIs. The most frequently selected features suggest working with a coach lowers injury likelihood, while following online training programs or self-created plans is positively associated with injury. Runners should also be cautious when using one interval training session each week, which tended to be linked to RRI, yet using a high frequency of interval training may offer protection against injury. The findings of this study should be interpreted with caution due to the issues associated with retrospective survey data. Future prospective observational and intervention-based research studies are needed to establish whether S&C activities reduce RRIs.

## Supporting information

S1 File Supplementary Material.docx. (DOCX)

## References

[1] Oja P, Titze S, Kokko S, Kujala UM, Heinonen A, Kelly P, et al. Health benefits of different sport disciplines for adults: systematic review of observational and intervention studies with meta-analysis. Br J Sports Med. 2015;49(7):434–40. doi: 10.1136/bjsports-2014-093885 25568330

[2] Oswald F, Campbell J, Williamson C, Richards J, Kelly P. A scoping review of the relationship between running and mental health. Int J Environ Res Public Health. 2020;17(21):8059. doi: 10.3390/ijerph17218059 33139666 PMC7663387

[3] Linton L, Valentin S. Running with injury: a study of UK novice and recreational runners and factors associated with running related injury. J Sci Med Sport. 2018;21(12):1221–5. doi: 10.1016/j.jsams.2018.05.021 29853263

[4] Clough PJ, Shepherd J, Maughan RJ. Marathon finishers and pre-race drop-outs. Br J Sports Med. 1989;23(2):97–101. doi: 10.1136/bjsm.23.2.97 2605449 PMC1478627

[5] Hespanhol Junior LC, van Mechelen W, Postuma E, Verhagen E. Health and economic burden of running-related injuries in runners training for an event: a prospective cohort study. Scand J Med Sci Sports. 2016;26(9):1091–9. doi: 10.1111/sms.12541 26282068

[6] Hulme A, Nielsen RO, Timpka T, Verhagen E, Finch C. Risk and protective factors for middle- and long-distance running-related injury. Sports Med. 2017;47:869–86. doi: 10.1007/s40279-016-0636-4 27785775

[7] Reece LJ, Owen K, Graney M, Jackson C, Shields M, Turner G, et al. Barriers to initiating and maintaining participation in parkrun. BMC Public Health. 2022;22(1):83. doi: 10.1186/s12889-022-12546-w 35027014 PMC8759213

[8] Stevinson C, Hickson M. Exploring the public health potential of a mass community participation event. J Public Health (Oxf). 2014;36(2):268–74. doi: 10.1093/pubmed/fdt082 23954885

[9] Liddle N, Taylor JM, Chesterton P, Atkinson G. The effects of exercise-based injury prevention programmes on injury risk in adult recreational athletes: a systematic review and meta-analysis. Sports Med. 2024;54(3):645–58. doi: 10.1007/s40279-023-01950-w 37889449

[10] Yeung SS, Yeung EW, Gillespie LD. Interventions for preventing lower limb soft-tissue running injuries. Cochrane Database Syst Rev. 2011;2011(7):CD001256. doi: 10.1002/14651858.CD001256.pub2 21735382 PMC13283770

[11] Kozinc Ž, Šarabon N. Effectiveness of movement therapy interventions and training modifications for preventing running injuries: a meta-analysis of randomized controlled trials. J Sports Sci Med. 2017;16(3):421–8. Available from: http://www.jssm.org 28912661 PMC5592295

[12] Wu H, Brooke-Wavell K, Fong DTP, Paquette MR, Blagrove RC. Do exercise-based prevention programs reduce injury in endurance runners? A systematic review and meta-analysis. Sports Med. 2024;54(5):1249–67. doi: 10.1007/s40279-024-01993-7 38261240 PMC11127851

[13] Blagrove RC, Brown N, Howatson G, Hayes PR. Strength and conditioning habits of competitive distance runners. J Strength Cond Res. 2020;34(5):1392–9. doi: 10.1519/JSC.0000000000002261 29023328

[14] Linton L, Valentin S. Running coaches and running group leaders’ engagement with, and beliefs and perceived barriers to prehabilitation and injury prevention strategies for runners. Phys Ther Sport. 2020;46:54–62. doi: 10.1016/j.ptsp.2020.08.004 32871363

[15] Van Eetvelde H, Mendonça LD, Ley C, Seil R, Tischer T. Machine learning methods in sport injury prediction and prevention: a systematic review. J Exp Orthop. 2021;8(1):27. doi: 10.1186/s40634-021-00346-x 33855647 PMC8046881

[16] Lillis C. Exploring machine learning, real-time bio-feedback, and inertial sensor accuracy for the prevention of running-related injuries. Dublin City University; 2022.

[17] Saarela M, Jauhiainen S. Comparison of feature importance measures as explanations for classification models. SN Appl Sci. 2021;3(2):272. doi: 10.1007/s42452-021-04148-9

[18] Lövdal SS, Den Hartigh RJR, Azzopardi G. Injury prediction in competitive runners with machine learning. Int J Sports Physiol Perform. 2021;16(10):1522–31. doi: 10.1123/ijspp.2020-0518 33931574

[19] Clarsen B, Myklebust G, Bahr R. Development and validation of a new method for the registration of overuse injuries in sports injury epidemiology: the Oslo Sports Trauma Research Centre (OSTRC) overuse injury questionnaire. Br J Sports Med. 2013;47(8):495–502. doi: 10.1136/bjsports-2012-091524 23038786

[20] Fragala MS, Cadore EL, Dorgo S, Izquierdo M, Kraemer WJ, Peterson MD. Resistance training for older adults: position statement from the national strength and conditioning association. J Strength Cond Res. 2019;33:2019–52. doi: 10.1519/JSC.0000000000003230 31343601

[21] Blagrove RC, Howatson G, Hayes PR. Effects of strength training on the physiological determinants of middle- and long-distance running performance: a systematic review. Sports Med. 2018;48:1117–49. doi: 10.1007/s40279-017-0835-7 29249083 PMC5889786

[22] Hübscher M, Zech A, Pfeifer K, Hänsel F, Vogt L, Banzer W. Neuromuscular training for sports injury prevention. Med Sci Sports Exerc. 2010;42(3):413–21. doi: 10.1249/mss.0b013e3181b88d37 19952811

[23] Teixeira RN, Lunardi A, da Silva RA, Lopes AD, Carvalho CRF. Prevalence of musculoskeletal pain in marathon runners who compete at the elite level. Int J Sports Phys Ther. 2016;11(1):126–31. 26900507 PMC4739041

[24] Hespanhol Junior LC, Costa LOP, Carvalho ACA, Lopes AD. A description of training characteristics and its association with previous musculoskeletal injuries in recreational runners: a cross-sectional study. Rev Bras Fisioter. 2012;16(1):46–53. doi: 10.1590/s1413-35552012005000005 22441228

[25] Van Middelkoop M, Kolkman J, Van Ochten J, Bierma-Zeinstra SMA, Koes B. Prevalence and incidence of lower extremity injuries in male marathon runners. Scand J Med Sci Sports. 2008;18(2):140–4. doi: 10.1111/j.1600-0838.2007.00683.x 17555538

[26] van Mechelen W, Hlobil H, Kemper HC, Voorn WJ, de Jongh HR. Prevention of running injuries by warm-up, cool-down, and stretching exercises. Am J Sports Med. 1993;21(5):711–9. doi: 10.1177/036354659302100513 8238713

[27] Sanfilippo D, Beaudart C, Gaillard A, Bornheim S, Bruyere O, Kaux J-F. What are the main risk factors for lower extremity running-related injuries? A retrospective survey based on 3669 respondents. Orthop J Sports Med. 2021;9(11):23259671211043444. doi: 10.1177/23259671211043444 34820458 PMC8606943

[28] Fredette A, Roy JS, Perreault K, Dupuis F, Napier C, Esculier JF. The association between running injuries and training parameters: a systematic review. J Athl Train. 2022;57:650–71. doi: 10.4085/1062-6050-0195.21 34478518 PMC9528699

[29] Hamstra-Wright KL, Coumbe-Lilley JE, Kim H, McFarland JA, Huxel Bliven KC. The influence of training and mental skills preparation on injury incidence and performance in marathon runners. J Strength Cond Res. 2013;27(10):2828–35. doi: 10.1519/JSC.0b013e31828a4733 23439344

[30] Hespanhol Junior LC, Pena Costa LO, Lopes AD. Previous injuries and some training characteristics predict running-related injuries in recreational runners: a prospective cohort study. J Physiother. 2013;59(4):263–9. doi: 10.1016/S1836-9553(13)70203-0 24287220

[31] Kemler E, Romeijn K, Vriend I, Huisstede B. The relationship between the use of running applications and running-related injuries. Phys Sportsmed. 2018;46(1):73–7. doi: 10.1080/00913847.2018.1412812 29202625

