# Supplementary material for: Associations between training behaviors and injuries in performance-oriented parkrunners using traditional statistical methods and machine learning
Source: PLoS One. 2026 Aug 19;21(8):e0350075. doi: 10.1371/journal.pone.0350075 (PMC13489425; doi:10.1371/journal.pone.0350075)
Supplement: Supplementary file 1 [file pone.0350075.s001.docx]

**Supplementary Material S1: Questionnaire Content**

**Information Section**

**Overuse injuries in the parkrun community: prevalence, consequences, and training causes**

Welcome and thank you for considering taking part in this online survey.

Before you decide whether to complete this survey, we would like you to understand why the research is being done and what it would involve for you. Please contact one of the investigators using the contact details below if you have any questions.

The purpose of this study is to gain information relating to the incidence and impact running-related overuse injury in the parkrun community. The study aims to (i) quantify the extent to which injuries prevent participation in running exercise and parkrun, (ii) describe how injury affects wellbeing, and (iii) examine whether exercise training factors predict injury occurrence in parkrun participants.

This study is part of a research project being run by Loughborough University. The study is being undertaken by Dr Richard Blagrove and Dr Clare Stevinson. You will be asked to complete an anonymous online survey, which should take no longer than 15 minutes to complete. You do not need to do anything before completing the survey. This is a low-risk activity and no disadvantages or risks have been identified in association with participating.

You must be over the age of 18 and have the capacity to fully understand and consent to this research.

Loughborough University will be using information/data from you to undertake this study and will act as the data controller for this study. No data will be passed or used by parkrun. This means that the University is responsible for looking after your information and using it properly.

The results of all responses will be analysed and written in scientific reports which may be published and/or presented at conferences. Data may also be used in student dissertations at Loughborough University. No individual will be identifiable in any report, presentation, or publication. All data will be stored on the password-protected University IT cloud-based storage. Only researchers directly involved in the study will have access to any of this information. Anonymised data will be retained electronically until the 1^st^ January 2027 and will then be deleted.

After you have read this information and asked any questions you may have, if you are happy to participate, please read the consent page and confirm your consent by checking the tick box at the bottom of the page. You can withdraw from the survey at any time by closing the browser. However, once you have submitted the survey it will not be possible to withdraw your data from the study.

**What personal information will be collected from me and how will it be used?**

Personal information is data that can be used to specifically identify you, such as your email, date of birth or medical history. There is an option to provide an email address at the end of this survey to be contacted about future research, however this is not mandatory. If you provide your email address it will not be passed to any other individuals or organisations outside of the research team involved with this study. The survey also contains information about your age, injury history, habitual exercise, and your height and body mass.

Under the General Data Protection Regulation (GDPR), some of the personal data which will be collected from you is categorised as “sensitive data”. The processing of this data is necessary for scientific research in accordance with safeguards. This means that study has gone through an ethical committee to ensure that the appropriate safeguards are put in place with respect to the use of your personal data. Personal data will be retained electronically until the 1^st^ January 2027 and will then be deleted. Personal data will be processed on the public task basis. For further details on the data protection legislation see: <https://ico.org.uk/your-data-matters/>

Dr Richard Blagrove (Responsible Investigator), School of Sport, Exercise and Health Sciences, Loughborough University, Loughborough, Leicestershire, LE11 3TU, R.C.Blagrove@lboro.ac.uk, 01509 226383

**What if I am not happy with how the research was conducted?**

If you are not happy with how the research was conducted, please contact the Secretary of the Ethics Review Sub-Committee, Research & Enterprise Office, Hazlerigg Building, Loughborough University, Epinal Way, Loughborough, LE11 3TU. Tel: 01509 222423. Email: [researchpolicy@lboro.ac.uk](mailto:researchpolicy@lboro.ac.uk)

The University also has policies relating to Research Misconduct and Whistle Blowing which are available online at <https://www.lboro.ac.uk/internal/research-ethics-integrity/research-integrity/> .

If you require any further information regarding the General Data Protection Regulations, please see: <https://www.lboro.ac.uk/privacy/research-privacy/>

**Informed Consent Section**

The purpose and details of this study have been explained to me.

I understand that this study is designed to further scientific knowledge and that all procedures have received a favourable decision from the Loughborough University Ethics Review Sub-Committee.

I have had an opportunity to ask questions about my participation.

I understand that I have the option of providing my email address only for the purposes of participating in future studies. Non-identifying demographic information will also be collected, e.g. sex, age.

I understand that I am under no obligation to take part in the study and can withdraw during the survey by closing the browser but will not be able to withdraw once my responses have been submitted.

I understand that information I provide will be used for a student’s dissertation.

I have read and understood the information section, and this informed consent section.

**Consent to Participate**

I voluntarily agree to take part in this study.

1. *I consent* (link to Section 1) */ do not consent* (link to final page)

**SECTION 1: Participant characteristics**

2. How old are you?

____ years

3. What was your assigned sex at birth?

Female / Male

4. What is your body mass (in kilograms)?

______ kg

5. What is your height?

______ cm

6. How many years have you been participating in parkrun for (if less than one year please use a fraction, e.g., 3 months = 0.25 years)?

______ years

7. How many parkruns have you completed?

_____

8. What is the fastest time you have completed a parkrun (or other 5 km run/race) in the last 12 months?

____ min ___ sec

9. How would you describe your level of participation in running?

Recreational - enjoyment and health reasons only (no target times)

Recreational – enjoyment and health reasons, plus to achieve a specific performance goal/time for myself

Competitive – races at local club level

Competitive – races at county or regional level

Competitive – races at national level

Competitive – races at international level

10. How long have you been running regularly for (at least once per week most weeks)?

Less than 6 months

6-12 months

1-2 years

2-5 years

5-10 years

>10 years

**SECTION 2: Exercise and training habits**

11. Over the last 12 months, how many times have you typically run each week?

1, 2, 3, 4, 5, 6, 7, 8 or more

12. Typically, how long do you run for each week in total (all runs combined)?

____ minutes

13. Typically, how many interval training running sessions do you perform each week?

0, 1, 2, 3, 4 or more

14. In the main, who has planned your running training over the last 12 months?

I have not had a training plan

I planned my own training

Followed an online or app-based training plan, eg ‘couch to 5k’

A running coach

Someone else (please specify)

15. Over the last 12 months, have you participated in any strength and conditioning activities on most weeks?

Yes / No

16. If yes, typically how many minutes and how often per week (sessions per week)?

| Activity | Yes/No | If yes, minutes per week | If yes, how often per week |
| --- | --- | --- | --- |
| Resistance training (i.e., exercises with barbell, dumbbells, kettlebells, machines, elastic bands, medicine balls) |  |  |  |
| Plyometrics (i.e., jumping, skipping, hopping, bounding) |  |  |  |
| Core stability (i.e., exercises that specifically target the trunk/abdominal region) |  |  |  |
| Bodyweight exercises (i.e., burpees, press-ups, lunges without weights) |  |  |  |
| Stretching or yoga |  |  |  |

17. For what reasons do you include the strength and conditioning activities listed above?

Improve general health (e.g. bone and joint health, prevent muscle mass loss, etc)

Improve running performance

Improve running technique

Reduce the risk of a previous injury returning

Reduce the risk of getting a new injury

Rehabilitation of an existing or ongoing injury

It is enjoyable

Improve body image

Other (please specify)

18. Other than running and the strength and conditioning activities (resistance training, plyometrics, bodyweight exercises, core stability exercises, stretching/yoga), have you participated in any other forms of exercise or sport most weeks over the last year?

Yes / No

19. If yes:

| Activity (please specify) | Minutes per week | How often per week |
| --- | --- | --- |
|  |  |  |
|  |  |  |
|  |  |  |
|  |  |  |
|  |  |  |

**SECTION 3: Muscle, joint and bone related problems and running**

20. Have you had any difficulties participating in normal running exercise and/or parkruns due to muscle, joint or bone related problems* at any point in the last 12 months?

**‘problems’ are defined as pain, aching, stiffness, looseness or other complaints.*

Yes / No

21. Have you had any difficulties participating in your normal running routine due to muscle, joint or bone related problems during the past week?

Full participation without problems

Full participation but with problems

Reduced participation due to problems

Cannot participate due to problems

22. To what extent have you reduced your running volume due to muscle, joint or bone related problems during the past week?

No reduction

To a minor extent

To a moderate extent

To a major extent

Cannot participate at all

23. Please indicate the location of the problem(s) you have experienced over the last 12 months and the number of days you were forced to reduce your normal running routine because of each problem:

| Location of the problem (drop-down menu: foot/toes, ankle/Achilles, calf/shin, knee, thigh, hip/groin, lower back, upper body/head) | Number of days the problem lasted | Did you visit a healthcare professional (e.g. physiotherapist), to obtain a diagnosis? |
| --- | --- | --- |
|  |  | Yes / No |
|  |  | Yes / No |
|  |  | Yes / No |
|  |  | Yes / No |
|  |  | Yes / No |

Add row (if required)

**SECTION 4: Psychological consequences of injury and parkrun identity**

24. Over the last 12 months, have you been forced to miss a parkrun (as a runner/walker) due to muscle, joint or bone related problem?

Yes / No

25. Over the last 12 months, how many parkrun events have you been forced to miss (as a runner/walker) due to muscle, joint or bone related problem?

_____ parkruns

26. A number of statements that describe how people feel when suffering a muscle, joint or bone problem that limits their participation in parkrun are given below. Please read each one and select the response which best describes how frequently you felt that way when suffering a problem that caused you to miss parkrun. Some statements describe positive feelings and some describe negative feelings. You may have experienced both positive and negative feelings at different times when missing parkrun.

|  | Never | Rarely | Sometimes | Often |
| --- | --- | --- | --- | --- |
| I felt dissatisfied with my life |  |  |  |  |
| I felt happy |  |  |  |  |
| I felt cheerless |  |  |  |  |
| I felt pleased with the way I am |  |  |  |  |
| I felt that life was enjoyable |  |  |  |  |
| I felt that life was meaningless |  |  |  |  |

27. Please read the parkrun identity statements below and indicate how strongly you agree or disagree with them.

|  | Strongly disagree | Disagree | Somewhat disagree | Neither agree nor disagree | Somewhat agree | Agree | Strongly agree |
| --- | --- | --- | --- | --- | --- | --- | --- |
| I consider myself a parkrunner |  |  |  |  |  |  |  |
| I have many goals related to parkrun |  |  |  |  |  |  |  |
| Most of my friends are parkrunners |  |  |  |  |  |  |  |
| parkrun is the most important part of my life |  |  |  |  |  |  |  |
| I spend more time thinking about parkrun than anything else |  |  |  |  |  |  |  |
| I need to participate in parkrun to feel good about myself |  |  |  |  |  |  |  |
| Other people see me mainly as a parkrunner |  |  |  |  |  |  |  |
| I feel bad about myself when I cannot do parkrun |  |  |  |  |  |  |  |
| parkrun is the only important thing in my life |  |  |  |  |  |  |  |
| I would be very depressed if I were injured and could not do parkrun |  |  |  |  |  |  |  |

**Closing section**

28. As a follow-up to this survey, researchers at Loughborough University are planning to run a study that aims to remotely monitor the exercise habits, injury incidence, and emotional impact of injury on runners over a 12-month period. Would you be interested in taking part in this follow-up study?

Yes / No

If yes, please provide an email address we can contact you on ___________

29. Would you like to be contacted via email regarding any other running-related studies that we are undertaking, including physiological laboratory testing of fitness?

Yes / No

If yes, please provide an email address we can contact you on ___________

Thank you for taking the time to look at our survey!

**Supplementary Material S2: Detailed Description of Data Cleaning Process**

Please note the difference between excluding rows and excluding answers. When a row is excluded, all of that participant’s responses are excluded. When an answer is excluded, the rest of the answers from the same participant are still kept.

- Exclude all **rows** where participant answer is “I do not consent” for Q1. I voluntarily agree to take part in this study.
- Exclude all **rows** where answer is <18 for Q2. How old are you?
- Inspect all **rows** where answer is >80 for Q2. How old are you? After inspection, one row was excluded because it reports age of 99 and filled in 5k performance time faster than the world record.
- Exclude all **rows** where participant answer is “Recreational without a focus on finishing times” for Q9. How would you describe your level of participation in running/walking?
- For Q12. Over the last 12 months, how many times have you typically run/walked each week? If the answer is >30, divide the answer by 52. If 10 ≤ answer ≤30, divide the answer by 7. This is to account for answers where the participant thought they were supposed to answer per month or per year. After the above calculations, round all answers to the nearest integer.
- For Q12. Over the last 12 months, how many times have you typically run/walked each week? After the above conversions, exclude all **rows** where the answer is <3.
- For Q13. Typically, how long (in minutes) do you run/walk as a means of exercise for each week in total (all runs/walks combined)? If the answer is <10, time the answer by 60 because the participant may have confused minutes with hours.
- If the value in Q13. Typically, how long (in minutes) do you run/walk as a means of exercise for each week in total (all runs/walks combined)? is less than the added value of Q20.1.b.-Q20.5.b. Activity: - How many minutes per week (in total)? Then the **row** is excluded because the sum of time doing sports other than running is more than running time for the participant.
- For Q4. What is your body mass in kilograms? (optional question) an upper limit is set to be the mean + 4SD and a lower limit is set to be 40kg. If the participant falls outside of the limits then the answer is excluded.
- For Q5. What is your height in centimetres? (optional question) all participants taller than 220cm (none) and all participants shorter than 140cm have their answers excluded.
- BMI is calculated from the above processed height and weight data.
- All participant answers with BMI >40 or <17.5 were inspected. One person reported an extremely large BMI (over 60) and reasonable 5k performance. The weight data of this person was excluded.
- For Q6. How long have you been participating in parkrun for? All years plus months answers was converted into months.
- For Q8. What is the fastest time you have completed a parkrun (or other 5 km run/race) in the last 12 months? Please enter time as mm:ss, for example 30 minutes 43 seconds = 30:43 all answers are converted into seconds. Numerous individual cases exist where participants did not fill in according to the rule (for instance, filling 3043 or 30;43 instead of 30:43). These were corrected manually on a case-by-case basis.
- For Q11. How many years have you been running/walking as a means of exercise regularly for (at least once per week most weeks)? If the answer is smaller than the answer to Q2. How old are you? by <6, the answer is excluded.
- For Q13. Typically, how long (in minutes) do you run/walk as a means of exercise for each week in total (all runs/walks combined)? All answers of 999 is excluded. 999 is the largest number fillable and is unlikely to be achieved every week. It is also >4SD above average.
- For Q14. Typically, how many interval sessions* do you perform each week? *an \'interval session\' involves alternating periods of fast and slow running/walking. All answers of “o” and “O” are changed into “0”.
- For Q17.1.b. Resistance training (i.e., exercises with barbell, dumbbells, kettlebells, machines, elastic bands, medicine balls) - If yes, how many minutes per week (in total) all answers <5 are excluded.
- For Q17.1.c. Resistance training (i.e., exercises with barbell, dumbbells, kettlebells, machines, elastic bands, medicine balls) - If yes, how often per week? All answers >10 are excluded.
- The above two steps are repeated for 17.2.b-17.5.b and 17.2.c-17.5.c.
- For Q20.1.a-20.5.a. Activity: - Please specify activity... answers are mapped based on their meanings. For instance, “Cycle”, “Cycling”, “bike”, “biking” are all mapped as “cycling”.
- For Q24.1.a-24.8.a. Problem: - Location of problem, Q24.1.b-24.8.b. Problem: - Approximate number of days the problem lasted, and Q24.1.c-24.8.c. Problem: - Did you visit a healthcare professional (e.g., physiotherapist), to obtain a diagnosis? Answers where the same location is filled in more than once are inspected and modified on a case-by-case basis. For instance, some people wrote “knee” 8 times and filled in the same days and whether a healthcare professional was visited for each one. This is apparently because the participant didn’t realize the looping is for multiple injuries. However, when the same location is filled in twice but the number of days are different, they are likely not referring to the same injury so the two numbers are added for that location.
- For Q24.1.b-24.8.b. Problem: - Approximate number of days the problem lasted, all numbers >365 are changed into 365.

**List of Available Descriptive Statistics**

Please note: count refers to the number of answers available. All non-available answers were either excluded or not provided by the participant.

Question: How old are you?

count	570

mean	52.45

std	12.41

min	19

25%	44

50%	54

75%	62

max	88

Question: What was your assigned sex at birth?

Male	293

Female	274

Prefer not to say	3

Question: What is your body mass in kilograms? (optional question)

count	504

mean	71.93

std	12.23

min	44

25%	63

50%	71

75%	80

max	110

Question: What is your height in centimetres? (optional question)

count	488

mean	172.34

std	9.03

min	146

25%	165

50%	173

75%	179

max	210

BMI (calculated from mass and height)

count	475

mean	24.25

std	3.44

min	17.28

25%	21.84

50%	23.81

75%	26.23

max	40.40

Question: How long have you been participating in parkrun for?

Note: unit is month

count	570

mean	65.86 (5.5 years)

std	42.42

min	1

25%	36

50%	60

75%	96

max	216 (18 years)

Question: How many parkruns have you completed?

count	570

mean	127.55

std	113.09

min	1

25%	35

50%	99

75%	188

max	594

Question: What is the fastest time you have completed a parkrun (or other 5 km run/race) in the last 12 months?

count	569

mean	1605.87 (26min 46s)

std	353.745371 (5min 54s)

min	900 (15min)

25%	1361 (22min 41s)

50%	1531 (25min 31s)

75%	1784 (29min 44s)

max	3609 (60min 9s)

Question: How would you describe your level of participation in running/walking?

Recreational with a focus on improving times	469

Competitive at club level	88

Competitive at county/regional level	7

Competitive at national level	5

Competitive at international level	1

Question: How many years have you been running/walking as a means of exercise regularly for (at least once per week most weeks)?

count	560

mean	14.72

std	13.89

min	0

25%	5

50%	10

75%	20

max	65

Question: Over the last 12 months, how many times have you typically run/walked each week?

3.0	214

4.0	149

5.0	95

6.0	54

7.0	50

8.0	7

9.0	1

Question: Typically, how long (in minutes) do you run/walk as a means of exercise for each week in total (all runs/walks combined)?

count	569.000000

mean	226.63 (3h 47min)

std	164.64 (2h 45min)

min	11

25%	120 (2h)

50%	180 (3h)

75%	300 (5h)

max	990 (16.5h)

Question: Typically, how many interval sessions* do you perform each week? *an \'interval session\' involves alternating periods of fast and slow running/walking.

1	269

0	204

2	66

3	14

4	11

5	4

7	1

6	1

Question: In the main, who has planned your running/walking training over the last 12 months?

I planned my own training	275

I have not had a training plan	179

Followed an online or app-based training plan, eg ‘couch to 5k’	66

A running coach	30

Other	20

Question: Over the last 12 months, have you participated in any strength and conditioning activities (i.e. resistance training, plyometrics, bodyweight exercises, core stability exercises, stretching/yoga) on most weeks?

Yes	371

No	199

Question: Resistance training (i.e., exercises with barbell, dumbbells, kettlebells, machines, elastic bands, medicine balls) - Yes/No

Yes	246

No	125

Question: Resistance training (i.e., exercises with barbell, dumbbells, kettlebells, machines, elastic bands, medicine balls) - If yes, how many minutes per week (in total)

count	235

mean	56.85

std	50.49

min	5

25%	25

50%	40

75%	70

max	360

Question: Resistance training (i.e., exercises with barbell, dumbbells, kettlebells, machines, elastic bands, medicine balls) - If yes, how often per week?

1.0	91

2.0	70

3.0	38

7.0	9

5.0	9

4.0	5

6.0	3

8.0	1

Question: Plyometrics (i.e., jumping, skipping, hopping, bounding) - Yes/No

No	309

Yes	62

Question: Plyometrics (i.e., jumping, skipping, hopping, bounding) - If yes, how many minutes per week (in total)

count	57

mean	26.82

std	28.29

min	5

25%	10

50%	20

75%	30

max	140

Question: Plyometrics (i.e., jumping, skipping, hopping, bounding) - If yes, how often per week?

1.0	37

2.0	12

3.0	6

0.0	3

7.0	2

5.0	1

Question: Core stability (i.e., exercises that specifically target the trunk/abdominal region) - Yes/No

Yes	251

No	120

Question: Core stability (i.e., exercises that specifically target the trunk/abdominal region) - If yes, how many minutes per week (in total)

count	235

mean	35.21

std	28.95

min	5

25%	15

50%	30

75%	45

max	180

Question: Core stability (i.e., exercises that specifically target the trunk/abdominal region) - If yes, how often per week?

1.0	102

2.0	55

3.0	29

4.0	11

5.0	9

7.0	8

6.0	4

0.0	1

Question: Bodyweight exercises (i.e., burpees, press-ups, lunges without weights) - Yes/No

Yes	199

No	172

Question: Bodyweight exercises (i.e., burpees, press-ups, lunges without weights) - If yes, how many minutes per week (in total)

count	185

mean	32.03

std	28.75

min	5

25%	15

50%	30

75%	35

max	210

Question: Bodyweight exercises (i.e., burpees, press-ups, lunges without weights) - If yes, how often per week?

1.0	71

2.0	53

3.0	23

4.0	14

5.0	6

7.0	6

6.0	4

10.0	2

0.0	1

Question: Stretching or yoga - Yes/No

Yes	274

No	97

Question: Stretching or yoga - If yes, how many minutes per week (in total)

count	254

mean	49.30

std	43.40

min	5

25%	20

50%	30

75%	60

max	300

Question: Stretching or yoga - If yes, how often per week?

1.0	64

3.0	53

2.0	42

7.0	24

4.0	22

6.0	19

5.0	13

10.0	3

Question: For what reasons do you include the strength and conditioning activity/activities listed above? (tick all that apply)

Improve general health (e.g. bone and joint health prevent muscle mass loss etc)	287

Reduce the risk of getting a new injury	236

Improve my running performance	214

Reduce the risk of a previous injury returning	213

I enjoy it	166

Rehabilitation of an existing or ongoing injury	162

Improve body image	99

Improve running technique	93

Other	11

Question: Other than running/walking and strength and conditioning activities (resistance training, plyometrics, bodyweight exercises, core stability exercises, stretching/yoga), have you participated in any other forms of exercise or sport most weeks over the last year?

No	363

Yes	207

Question: Activity: - Please specify activity...

cycling	93

swimming	70

tennis	8

football	7

spinning	6

badminton	5

netball	4

golf	3

crosstrainer	3

rowing	3

walking	3

dance	2

taekwondo	2

squash	2

hockey	2

karate	2

kayaking	2

cricket	2

aerobics	2

orienteering	2

dancefit	1

sup	1

paddleboarding	1

zumba	1

boxing	1

walkingrugby	1

tabletennis	1

massages	1

trampolining	1

bouldering	1

hillwalking	1

bodycombat	1

pilates	1

poledancing	1

dancing	1

climbing	1

snowboard	1

caving	1

ballet	1

horseriding	1

kungfu	1

elliptical	1

waterskiing	1

gym	1

kayak	1

aquajog	1

cardiomachines	1

racquetball	1

Minutes per week doing cycling for those who filled in cycling and filled in minutes per week for cycling.

Count: 79

Mean: 80.37

Std: 47.60

Max: 300

Min: 15

Sessions per week doing cycling for those who filled in cycling and filled in sessions per week for cycling.

count	81

mean	1.86

std	1.03

min	1

25%	1

50%	2

75%	2

max	5

Minutes per week doing swimming for those who filled in swimming and filled in minutes per week for swimming.

Count: 63

Mean: 64.52

Std: 38.54

Max: 210

Min: 20

Sessions per week doing swimming for those who filled in swimming and filled in sessions per week for swimming.

count	64

mean	1.75

std	0.94

min	1

25%	1

50%	2

75%	2

max	6

Question: Have you had any difficulties participating in normal running/walking exercise and/or parkrun due to muscle, joint or bone related problems* at any point in the last 12 months? *‘problems’ are defined as pain, aching, stiffness, looseness or other complaints.

Yes	483

No	84

Question: Have you had any difficulties participating in your normal running/walking exercise routine due to muscle, joint or bone related problems during the past week?

Reduced participation due to problems	189

Full participation without problems	122

Full participation but with problems	98

Cannot participate due to problems	77

Question: To what extent have you reduced your running/walking exercise volume due to muscle, joint or bone related problems during the past week?

No reduction	148

To a moderate extent	102

To a major extent	100

To a minor extent	95

Cannot participate at all	41

Question: Please indicate the location of the problem(s) you have experienced over the last 12 months and the number of days you were forced to reduce your normal running/walking exercise routine because of each problem. Problem: - Location of problem

knee	167

ankle/achilles tendon	151

calf/shin	118

hip/groin	110

foot/toes	84

lower back	78

thigh (between hip and knee)	75

upper body and head	19

Knee: number of days forced to reduce normal running/walking

count	147

mean	99.67

std	117.31

min	1

25%	20

50%	60

75%	120

max	365

Knee: Whether a diagnosis was obtained

Yes	76

No	74

Ankle/achilles tendon: number of days forced to reduce normal running/walking

count	133

mean	86.47

std	102.11

min	0

25%	21

50%	45

75%	100

max	365

Ankle/achilles tendon: Whether a diagnosis was obtained

No	72

Yes	60

Calf/shin: number of days forced to reduce normal running/walking

count	104

mean	40.48

std	55.49

min	2

25%	14

50%	21

75%	45

max	365

Calf/shin: Whether a diagnosis was obtained

No	63

Yes	43

Hip/groin: number of days forced to reduce normal running/walking

count	102

mean	74.63

std	92.69

min	2

25%	14

50%	38

75%	90

max	365

Hip/groin: Whether a diagnosis was obtained

Yes	60

No	42

Foot/toes: number of days forced to reduce normal running/walking

count	81

mean	106.02

std	114.76

min	0

25%	21

50%	60

75%	150

max	365

Foot/toes: Whether a diagnosis was obtained

Yes	54

No	26

Lower back: number of days forced to reduce normal running/walking

count	64

mean	71.36

std	113.48

min	2

25%	9.25

50%	17.50

75%	60

max	365

Lower back: Whether a diagnosis was obtained

Yes	34

No	34

Thigh: number of days forced to reduce normal running/walking

count	65

mean	64.98

std	88.48

min	1

25%	14

50%	21

75%	70

max	365

Thigh: Whether a diagnosis was obtained

No	38

Yes	27

Upper body and head: number of days forced to reduce normal running/walking

count	19

mean	33.53

std	35.17

min	1

25%	8.50

50%	21

75%	45

max	120

Upper body and head: Whether a diagnosis was obtained

Yes	12

No	7

Question: As a runner/walker, over the last 12 months, have you been forced to miss a parkrun event due to a muscle, joint or bone related problem?

Yes	362

No	124

SC minutes total: The total number of minutes a person participated in any S&C activity (as listed in questions above) per week during the past 12 months. If there were no participation, the number is 0.

count	570

mean	73.00

std	95.65

min	0

25%	0

50%	46.50

75%	120

max	790

Lower limb days total: The total number of days a person’s normal running/walking is reduced due to a lower limb problem (knee, ankle/achilles, calf/shin, foot/toes, thigh) during the past 12 months.

count	570

mean	89.10

std	137.17

min	0

25%	0

50%	31

75%	100

max	1095

Lower limb injuries: Whether a person experienced any lower limb injuries during the past 12 months.

yes	416

no	154

**Supplementary Material S3: Workflow Diagram**

**Supplementary Material S4: List of features**

**Features Selected for Each Classifier**

| Classifier | Feature Indexes |
| --- | --- |
| Decision Tree | 70, 31, 82, 80, 15, 38 |
| Random Forest | 5, 31, 80, 6, 34, 70, 30, 79, 81, 73, 23 |
| SVM | 5, 6, 31, 80, 34, 70, 30, 79, 73, 23, 81 |
| KNN | 5, 6, 31, 80, 34, 70, 30, 79, 73, 23, 81 |
| Naïve Bayes | 5, 6, 31, 80, 34, 70, 30, 79, 73, 23, 81, 15, 64, 60 |
| Adaboost | 5, 6, 85, 86, 79, 16, 87, 17, 88 |
| Gradient Boosting | 70, 80, 31, 5, 81, 15 |
| Multilayer Perceptron | 5, 6, 31, 80, 34, 70, 30, 79, 73, 23, 81, 15 |

Feature indexes for the best feature subset by which each classifier yielded the highest average AUC. See below for the feature name corresponding to each feature index.

| **Index** | **Column Name** |
| --- | --- |
| 0 | 2. How old are you? |
| 1 | 3. What was your assigned sex at birth? |
| 2 | 4. What is your body mass in kilograms? (optional question) |
| 3 | 5. What is your height in centimetres? (optional question) |
| 4 | 6. How long have you been participating in parkrun for? |
| 5 | 7. How many parkruns have you completed? |
| 6 | 8. What is the fastest time you have completed a parkrun (or other 5 km run/race) in the last 12 months? Please enter time as mm:ss, for example 30 minutes 43 seconds = 30:43 |
| 7 | 11. How many years have you been running/walking as a means of exercise regularly for (at least once per week most weeks)? |
| 8 | 13. Typically, how long (in minutes) do you run/walk as a means of exercise for each week in total (all runs/walks combined)? |
| 9 | 16. Over the last 12 months, have you participated in any strength and conditioning activities (i.e. resistance training, plyometrics, bodyweight exercises, core stability exercises, stretching/yoga) on most weeks? |
| 10 | 17.1.b. Resistance training (i.e., exercises with barbell, dumbbells, kettlebells, machines, elastic bands, medicine balls) - If yes, how many minutes per week (in total) |
| 11 | 17.2.b. Plyometrics (i.e., jumping, skipping, hopping, bounding) - If yes, how many minutes per week (in total) |
| 12 | 17.3.b. Core stability (i.e., exercises that specifically target the trunk/abdominal region) - If yes, how many minutes per week (in total) |
| 13 | 17.4.b. Bodyweight exercises (i.e., burpees, press-ups, lunges without weights) - If yes, how many minutes per week (in total) |
| 14 | 17.5.b. Stretching or yoga - If yes, how many minutes per week (in total) |
| 15 | 19. Other than running/walking and strength and conditioning activities (resistance training, plyometrics, bodyweight exercises, core stability exercises, stretching/yoga), have you participated in any other forms of exercise or sport most weeks over the last year? |
| 16 | BMI |
| 17 | SC_minutes_total |
| 18 | 9. How would you describe your level of participation in running/walking?_1 |
| 19 | 9. How would you describe your level of participation in running/walking?_2 |
| 20 | 9. How would you describe your level of participation in running/walking?_3 |
| 21 | 9. How would you describe your level of participation in running/walking?_4 |
| 22 | 9. How would you describe your level of participation in running/walking?_5 |
| 23 | 12. Over the last 12 months, how many times have you typically run/walked each week?_3 |
| 24 | 12. Over the last 12 months, how many times have you typically run/walked each week?_4 |
| 25 | 12. Over the last 12 months, how many times have you typically run/walked each week?_5 |
| 26 | 12. Over the last 12 months, how many times have you typically run/walked each week?_6 |
| 27 | 12. Over the last 12 months, how many times have you typically run/walked each week?_7 |
| 28 | 12. Over the last 12 months, how many times have you typically run/walked each week?_8 |
| 29 | 12. Over the last 12 months, how many times have you typically run/walked each week?_9 |
| 30 | 14. Typically, how many interval sessions* do you perform each week? *an 'interval session' involves alternating periods of fast and slow running/walking._0 |
| 31 | 14. Typically, how many interval sessions* do you perform each week? *an 'interval session' involves alternating periods of fast and slow running/walking._1 |
| 32 | 14. Typically, how many interval sessions* do you perform each week? *an 'interval session' involves alternating periods of fast and slow running/walking._2 |
| 33 | 14. Typically, how many interval sessions* do you perform each week? *an 'interval session' involves alternating periods of fast and slow running/walking._3 |
| 34 | 14. Typically, how many interval sessions* do you perform each week? *an 'interval session' involves alternating periods of fast and slow running/walking._4 |
| 35 | 14. Typically, how many interval sessions* do you perform each week? *an 'interval session' involves alternating periods of fast and slow running/walking._5 |
| 36 | 14. Typically, how many interval sessions* do you perform each week? *an 'interval session' involves alternating periods of fast and slow running/walking._6 |
| 37 | 14. Typically, how many interval sessions* do you perform each week? *an 'interval session' involves alternating periods of fast and slow running/walking._7 |
| 38 | 17.1.c. Resistance training (i.e., exercises with barbell, dumbbells, kettlebells, machines, elastic bands, medicine balls) - If yes, how often per week?_0.0 |
| 39 | 17.1.c. Resistance training (i.e., exercises with barbell, dumbbells, kettlebells, machines, elastic bands, medicine balls) - If yes, how often per week?_1.0 |
| 40 | 17.1.c. Resistance training (i.e., exercises with barbell, dumbbells, kettlebells, machines, elastic bands, medicine balls) - If yes, how often per week?_2.0 |
| 41 | 17.1.c. Resistance training (i.e., exercises with barbell, dumbbells, kettlebells, machines, elastic bands, medicine balls) - If yes, how often per week?_3.0 |
| 42 | 17.1.c. Resistance training (i.e., exercises with barbell, dumbbells, kettlebells, machines, elastic bands, medicine balls) - If yes, how often per week?_4.0 |
| 43 | 17.1.c. Resistance training (i.e., exercises with barbell, dumbbells, kettlebells, machines, elastic bands, medicine balls) - If yes, how often per week?_5.0 |
| 44 | 17.1.c. Resistance training (i.e., exercises with barbell, dumbbells, kettlebells, machines, elastic bands, medicine balls) - If yes, how often per week?_6.0 |
| 45 | 17.1.c. Resistance training (i.e., exercises with barbell, dumbbells, kettlebells, machines, elastic bands, medicine balls) - If yes, how often per week?_7.0 |
| 46 | 17.1.c. Resistance training (i.e., exercises with barbell, dumbbells, kettlebells, machines, elastic bands, medicine balls) - If yes, how often per week?_8.0 |
| 47 | 17.2.c. Plyometrics (i.e., jumping, skipping, hopping, bounding) - If yes, how often per week?_0.0 |
| 48 | 17.2.c. Plyometrics (i.e., jumping, skipping, hopping, bounding) - If yes, how often per week?_1.0 |
| 49 | 17.2.c. Plyometrics (i.e., jumping, skipping, hopping, bounding) - If yes, how often per week?_2.0 |
| 50 | 17.2.c. Plyometrics (i.e., jumping, skipping, hopping, bounding) - If yes, how often per week?_3.0 |
| 51 | 17.2.c. Plyometrics (i.e., jumping, skipping, hopping, bounding) - If yes, how often per week?_5.0 |
| 52 | 17.2.c. Plyometrics (i.e., jumping, skipping, hopping, bounding) - If yes, how often per week?_7.0 |
| 53 | 17.3.c. Core stability (i.e., exercises that specifically target the trunk/abdominal region) - If yes, how often per week?_0.0 |
| 54 | 17.3.c. Core stability (i.e., exercises that specifically target the trunk/abdominal region) - If yes, how often per week?_1.0 |
| 55 | 17.3.c. Core stability (i.e., exercises that specifically target the trunk/abdominal region) - If yes, how often per week?_2.0 |
| 56 | 17.3.c. Core stability (i.e., exercises that specifically target the trunk/abdominal region) - If yes, how often per week?_3.0 |
| 57 | 17.3.c. Core stability (i.e., exercises that specifically target the trunk/abdominal region) - If yes, how often per week?_4.0 |
| 58 | 17.3.c. Core stability (i.e., exercises that specifically target the trunk/abdominal region) - If yes, how often per week?_5.0 |
| 59 | 17.3.c. Core stability (i.e., exercises that specifically target the trunk/abdominal region) - If yes, how often per week?_6.0 |
| 60 | 17.3.c. Core stability (i.e., exercises that specifically target the trunk/abdominal region) - If yes, how often per week?_7.0 |
| 61 | 17.4.c. Bodyweight exercises (i.e., burpees, press-ups, lunges without weights) - If yes, how often per week?_0.0 |
| 62 | 17.4.c. Bodyweight exercises (i.e., burpees, press-ups, lunges without weights) - If yes, how often per week?_1.0 |
| 63 | 17.4.c. Bodyweight exercises (i.e., burpees, press-ups, lunges without weights) - If yes, how often per week?_2.0 |
| 64 | 17.4.c. Bodyweight exercises (i.e., burpees, press-ups, lunges without weights) - If yes, how often per week?_3.0 |
| 65 | 17.4.c. Bodyweight exercises (i.e., burpees, press-ups, lunges without weights) - If yes, how often per week?_4.0 |
| 66 | 17.4.c. Bodyweight exercises (i.e., burpees, press-ups, lunges without weights) - If yes, how often per week?_5.0 |
| 67 | 17.4.c. Bodyweight exercises (i.e., burpees, press-ups, lunges without weights) - If yes, how often per week?_6.0 |
| 68 | 17.4.c. Bodyweight exercises (i.e., burpees, press-ups, lunges without weights) - If yes, how often per week?_7.0 |
| 69 | 17.4.c. Bodyweight exercises (i.e., burpees, press-ups, lunges without weights) - If yes, how often per week?_10.0 |
| 70 | 17.5.c. Stretching or yoga - If yes, how often per week?_0.0 |
| 71 | 17.5.c. Stretching or yoga - If yes, how often per week?_1.0 |
| 72 | 17.5.c. Stretching or yoga - If yes, how often per week?_2.0 |
| 73 | 17.5.c. Stretching or yoga - If yes, how often per week?_3.0 |
| 74 | 17.5.c. Stretching or yoga - If yes, how often per week?_4.0 |
| 75 | 17.5.c. Stretching or yoga - If yes, how often per week?_5.0 |
| 76 | 17.5.c. Stretching or yoga - If yes, how often per week?_6.0 |
| 77 | 17.5.c. Stretching or yoga - If yes, how often per week?_7.0 |
| 78 | 17.5.c. Stretching or yoga - If yes, how often per week?_10.0 |
| 79 | Planning_personnel_A running coach |
| 80 | Planning_personnel_Followed an online or app-based training plan, eg ‘couch to 5k’ |
| 81 | Planning_personnel_I have not had a training plan |
| 82 | Planning_personnel_I planned my own training |
| 83 | Planning_personnel_Other |
| 84 | lower_limb_injuries |
|  |  |
| 85 | 9. How would you describe your level of participation in running/walking? |
| 86 | 14. Typically, how many interval sessions* do you perform each week? *an 'interval session' involves alternating periods of fast and slow running/walking. |
| 87 | 17.4.c. Bodyweight exercises (i.e., burpees, press-ups, lunges without weights) - If yes, how often per week? |
| 88 | 17.2.c. Plyometrics (i.e., jumping, skipping, hopping, bounding) - If yes, how often per week? |

**Supplementary Material S5: Descriptions of Feature Selection Methods**

Principle Component Analysis (PCA)

PCA maps all data points in a multi-dimensional feature space and then looks for a line that represents the best fit of the data points in the feature space by minimising squared distances between data points and the line. This initial line is the first principle component (PC1). The second principle component is created by looking for the best fit line perpendicular to PC1 in the feature space (PC2). PC1 and PC2 together creates a 2-dimensional plane for which data points can be mapped against. PC3 is the best fit line perpendicular to this 2-dimensional plane, and so on. An eigenvector denotes the direction of a principle component, while an eigenvalue denotes the variance which is the average of the sum of square of the distances between mapped data points and the average. The PCA calculation runs as follows:

- Standardisation:

Each feature *X_i_* is standardized to have a mean of 0 and a standard deviation of 1. The standardized value *Z_i_* is calculated as:

where *μ_i_* is the mean and *σ_i_* is the standard deviation of feature *X_i_.*

- Computation of Covariance Matrix:

For a dataset with n features, the covariance matrix Σ is an *n×n* matrix where each element Σ*_ij_* is the covariance between feature *i* and feature *j*. The covariance is calculated as:

where *N* is the number of data points.

- Eigenvalue and Eigenvector Calculation:

Where λ*_i_* and v*_i_* are the *i*-th eigenvalue and eigenvector of Σ, respectively.

- Principle Component Selection

Principle components are selected by ranking eigenvalues in descending orders and selecting the corresponding eigenvectors. Each resultant PC is in the form of a linear equation:

where a*_ij_* are the elements of the *i*-th eigenvector.

In the current experiment, the top 15 PCs were selected and were tested on each ML classifier in descending orders of eigenvalues (i.e. PC1, PC1+PC2, PC1+PC2+PC3, etc.).

Decision Tree Classifier

Decision tree classifier can also be used to rank feature importance apart from serving as an ML classifier. The ranking is based on a certain feature’s ability to minimise the Gini index. For Gini index calculation, please see the next section (Descriptions of Machine Learning Classifiers). The ability for feature *f* to reduce Gini index at node *t* is calculated as:

Where *ΔI* is the Gini index reduction value; *N*_parent_(*t*), *N*_left_(*t*), and *N*_right_(*t*) are the number of samples in the parent node and each of the children nodes; *I*_parent_(*t*), *I*_left_(*t*), and *I*_right_(*t*) are the Gini indexes at respective nodes.

Total impurity decrease that a feature provides is the addtion of all *ΔI*s where that feature is used as division rule. Feature importance is ranked by ranking features’ total impurity decreases.

Relief

Relief calculates feature importance by updating a feature weight parameter. It recursively takes random samples from the dataset, finds the nearest hit and the nearest miss based on each target feature, then updates the feature weight parameter depending on how close the nearest hit is and how far the nearest miss is to the sample regarding the target feature. The calculation procedure is as follows:

For each feature weight W[*i*] (initialised to 0 at the beginning of calculation):

Where *diff*(*x*, *y*) is a function calculating the difference between *x* and *y*, R[*i*] is the value of feature *i* of a randomly selected sample, H[*i*] is the value of feature *i* for the nearest hit (sample that has the same outcome as R and the closes value for *i* compared to R), M[*i*] is the value of feature *i* for the nearest miss (sample that has the opposite outcome relative to R and has the closest value for *i* compared to R), and *m* represents the number of samples selected for calculation.

As the sample size for the current study is relatively small and thus does not induce excessive computational cost, *m* is set to be the number of samples in the entire dataset (n=570) to maximise accuracy.

Least Absolute Shrinkage and Selection Operator (LASSO)

LASSO increases bias and reduces variance of the prediction by introducing a penalty score. The penalty score is calculated as the absolute value of the target coefficient times a tuning parameter. By adjusting this tuning parameter, some less important coefficients will be reduced to 0, dropping the corresponding variable/feature. The larger the tuning parameter is set, the more coefficients get reduced to 0, meaning less features get retained. The optimal tuning parameter value is usually determined through practice (trying out which value produces the best result). For a dataset with *n* data points and *p* features, the LASSO calculation is as follows:

Where β_0_ to β*_p_* are the coefficients of the model, y*_i_* is the outcome, x*_ij_* are the predictor variables corresponding to each feature, and λ is the penalty parameter.

In the current experiment, α value corresponds to λ value in the above equation. Features were selected by adjusting α value and recursively testing selected features based on rank order of coefficient values.

**Supplementary Material S6: Descriptions of Machine learning Classifiers**

Decision Tree

Decision tree is a simple form of classifier where the samples are split by decision rules based on feature values. The decision rules are determined by calculating and comparing the performance of each possible splitting method (splitting by categories for categorical variables and splitting by different possible values for continuous variables). Two ways of performance calculations (generally the two methods yield similar performances) are information gain based on entropy and the Gini index. Larger information gain and lower Gini index correspond to better node performance. The python code used in the current experiment uses Gini index by default. The calculation for Gini index is as follows:

Where *S* is the set of data points in the target node after splitting, and *p_i_* is the number of data points in class *i* divided by the number of data points in *S*. The weighed average of the Gini indexes for all children nodes (nodes after splitting) is used as the performance metric for the corresponding decision rule.

Random Forest

Random forest classifier uses multiple decision trees to perform learning independently. When making predictions, the target sample is passed through all trained decision trees, and a majority vote among the results is used to determine the final prediction. With each decision tree, a bootstrap sample is created by randomly taking samples fromm the original dataset with replacement, resulting in a different bootstrap sample for each tree. In the current experiment, each bootstrap sample is set to contain the same amount of samples as the original dataset.

Support Vector Machine (SVM)

SVM classifier transforms the feature space into a higher dimensional space where it is more likely that the data can be separated linearly. The kernel trick can be used in SVM to calculate extent of similarities between each two data points without having to calculate the separating hyperplane, saving computational resources. In the current study, the default setting during feature selection and the best-performing hyperparameter set both used the Radial Basis Function (RBF), which uses the kernel trick to perform calculations as if data is transformed into a space with infinite dimensions. In addition, soft margin and slack variables are introduced to allow some misclassifications as real-life data is usually not perfectly separable.

The RBF kernel between x*_i_* and x*_j_* is:

Where γ determines the width of the decision boundary, i.e. the extent to which points far away will be accounted for in the calculation.

For an RBF function, the optimisation problem is formulated as:

Subject to:

Where α are the Lagrange multipliers, and y are the class labels (outcomes), and *C* is the regularisation parameter that controls the trade-off between the slack variable penalty and the margin size.

After training, prediction for new data point x is made based on the decision function:

Where *b* is a bias term calculated as:

*b* is averaged among all support vectors to provide numerical stability.

K Nearest Neighbour (KNN)

KNN classifier classifies each data point by taking the majority vote from its K number of nearest data points. In the current study, the feature selection process uses uniform weights as default, meaning that votes from nearest data points are considered equally. The hyperparameter tuning process selected distance-based weighing as the better-performing hyperparameter, in which case weighing of a neighbor point is inversely proportional to the target data point.

Naive Bayes

Naive Bayes classifier is based upon the Bayes’ theorem which is mathematically stated as:

Naive Bayes classifier assumes the probability of every feature to be independent of the probability of all other features. Under this assumption, the probability of outcome *C* for a set of features X*_1_* to X*_n_* is:

As the denominator is constant for both classes, predictions can be made by comparing the numerators between probability calculations for the two outcomes (injured/uninjured). In the current experiment, as some of the features are continuous variables, Gaussian Naive Bayes is used which assumes that all continuous variables follow the Gaussian distribution.

Adaboost

Adaboost uses a large number of weak classifiers (single-node decision trees) in sequential order and update weights for the classifier and for correctly/incorrectly classified samples based on results form the previously used classifier. Final predictions are made by taking votes from each weak classifier based on their assigned weights. Each weak learner is selected via minimising the Gini index as described in the decision tree classifier section above. After training on the initial weak classifier, for weak learner *i* and sample *j*, the error rate ε*_i_* is updated as :

Where *D_i_* is the current weight of each sample; II(Condition) is an indicator function which returns 1 or 0 when the condition is true or false; y*_j_* is the outcome, and *h_i_*(x*_j_*) is the predicted outcome.

The weak learner’s weight α*_i_* is calculated as:

For the next weak classifier, sample weights *D_i_*_+1_(*j*) is:

Where *Z_i_* is a normalisation fatctor to convert the new sample weights into a probability distribution (sum of all weights equals 1).

For predictions, all weak learners vote with their assigned weights, and the prediction *H*(*x*) is given as:

Where *T* is the total number of weak learners.

Gradient Boosting

Gradient boosting classifier also uses a number of weak learners to make predictions. However, it is different than Adaboost in that: 1. The weak learners it uses can be decision trees with multiple nodes rather than a single node. 2. Rather than letting the weak learners each make a prediction and take votes, each gradient boosting weak learner try to shift the log odds of samples in each decision tree leaf a little closer to their actual outcome, and the extent of shift is determined by a customised learning rate. With each weak classifier, gradient boosting classifier aims to minimise the loss function *L* given as:

Where *y* is the outcome and *F*(*x*) is the prediction.

Each weak learner is created through the same process as in decision tree classifier by minimising the Gini index, however the maximum depth of the tree is limited to limit the complexity of each weak classifier. For a maximum of *M* iterations, the predictive model *F_m_*(*x*) at each iteration *m* is given as:

Where *F_m-1_*(*x*) is the model after previous iteration, *η* is the customised learning rate, *γ_m_* is the weight assigned to the *m*^th^ weak learner, and *h_m_*(*x*) is the current decision tree.

When creating the decision tree, the classifier performs gradient descent on the loss function to find the best model. In other words, each weak learner is fitted based on the negative gradient of the loss function, given as:

The final prediction function *F_M_*(*x*) is given as:

The final log odds relative to a log odds threshold is used to determine the prediction.

Multilayer Perceptron (MLP)

MLP is a type of neuro-network consisting of an input layer, an output layer, and multiple hidden node layers. The input layer is used for feature input, while the output layer gives the prediction, which in the currently study is injured/uninjured classification. Each node in a hidden layer takes information from all nodes from the previous layer and performs a calculation based on input weights and bias terms. The input weights and bias terms are then updated via backpropagation, which is a process of employing gradient descent on the loss function for each training sample from the result backward through the hidden layers, one layer at a time.

The forward propagation function (i.e. the calculation of prediction based on input features) is given as:

Where *a_j_*^(^*^l^*^)^ is the activation of the *j*^th^ neuron in the *l*^th^ layer, *w_ji_*^(^*^l^*^)^ is the weight carried forward by the *i*^th^ neuron in the (*l*-1)^th^ layer to the *j*^th^ neuron in the *l*^th^ layer, *b_j_*^(^*^l^*^)^ is the bias term corresponding to the weight, and *f* is the activation function.

During the feature selection process, Rectified Linear Unit (ReLU) was used as the default activation function, which is given as:

This function outputs *x* as long as *x* is greater than 0.

During hyperparameter tuning, hyperbolic tangent (tanh) was selected as the best-performing activation function, which is given as:

Backpropagation is used to update weights and bias terms for each node relative to nodes in the previous layer. The gradient of the loss for layer *l* is given as:

The updated weight *w* is:

Where η is the customised learning rate.

The bias term *b* is updated through the same gradient descent process.

**Supplementary Material S7: Description of Shapley Additive Explanations (SHAP)**

SHAP is a method to explain feature contributions to ML models based on game theory. The underlying principle is to take away different combinations of features (including the target feature) and see how the model prediction varies. To calculate all possible feature combinations for all features can be computationally intensive, so the Kernel Explainer is often used, which uses linear regression to approximate the SHAP values. The SHAP values are estimated by solving:

Where *S* is the subset of features, *N* is the set of all features, *f_x_*(*S*) is the model’s prediction using sebset *S*, *φ*_0_ is the model’s prediction with no features, *φ_i_* is the target SHAP value, and *w*(*S*) is a weighed term based on the number of features present in *S*.

The SHAP summary plot contains the SHAP values for each feature and each instance. It shows whether high/low feature value has a positive influence or negative influence on the final predicted outcome.

**Supplementary Material S8: Optimisation Results**

|  | LASSO-No one hot coding | Relief-N | Decision Tree-N | PCA-N | PCA-Y | Decision Tree-Y | Relief-Y | LASSO-Y |
| --- | --- | --- | --- | --- | --- | --- | --- | --- |
| Decision Tree-AUC | 0.591646825 | 0.577262873 | 0.541283391 | 0.551845238 | 0.57434669 | 0.536872096 | 0.585044038 | 0.607582511 |
| Random Forest-AUC | 0.648128871 | 0.614460414 | 0.60304128 | 0.615958914 | 0.55692049 | 0.60898495 | 0.619050039 | 0.666123451 |
| SVM-AUC | 0.603896148 | 0.581404859 | 0.627574042 | 0.600471835 | 0.583591754 | 0.599380565 | 0.570989644 | 0.640121951 |
| KNN-AUC | 0.629741338 | 0.564866434 | 0.575122919 | 0.595428039 | 0.570792441 | 0.56778915 | 0.581286053 | 0.639396293 |
| Naive Bayes-AUC | 0.638019744 | 0.600316976 | 0.603616918 | 0.594105207 | 0.586832172 | 0.597115273 | 0.596490031 | 0.658463995 |
| Adaboost-AUC | 0.631613192 | 0.614025358 | 0.590389082 | 0.579204413 | 0.56008493 | 0.578411731 | 0.607204559 | 0.625324477 |
| Gradient Boosting-AUC | 0.632443138 | 0.594613337 | 0.581214915 | 0.602241096 | 0.56123766 | 0.561546167 | 0.58543554 | 0.642285134 |
| MLP-AUC | 0.646279036 | 0.581117886 | 0.611221932 | 0.610096303 | 0.612425474 | 0.625161634 | 0.611633275 | 0.657875048 |
| Decision Tree-features | 27, 12, 23, 29, 28 | 29, 12, 28, 11, 1 | 6, 5, 2, 0, 8, 24, 10, 3, 25, 4, 14, 9, 18, 28, 20, 26, 17, 23, 13, 22, 29, 16, 19, 11, 1, 27 |  |  | 6, 16, 5, 7, 2, 8, 0, 3, 4, 12, 79, 81, 13, 17, 10, 25, 70, 40, 24, 30, 76, 33, 43, 22, 9 | 31, 9, 30, 82, 70 | 70, 31, 82, 80, 15, 38 |
| Random Forest-features | 5, 6, 27, 26, 12, 11, 23, 22, 28 | 29, 12, 28, 11, 1, 17, 13, 23, 25, 5, 6, 9, 7 | 6, 5, 2, 0, 8, 24, 10, 3, 25, 4, 14, 9, 18, 28, 20, 26, 17, 23 |  |  | 6, 16, 5, 7, 2, 8, 0, 3, 4, 12, 79, 81, 13, 17, 10, 25, 70, 40, 24, 30, 76, 33, 43, 22 | 31, 9, 30, 82, 70, 38, 81, 53, 23, 1, 6, 18, 61, 17, 5, 15, 32, 19, 54, 39, 24, 2, 10 | 5, 31, 80, 6, 34, 70, 30, 79, 81, 73, 23 |
| SVM-features | 6, 5, 7, 11, 26, 24, 22, 19, 15, 4, 25, 9, 27, 0, 12, 21, 29 | 29, 12, 28, 11, 1, 17, 13, 23, 25, 5, 6, 9, 7, 19, 21, 27, 2, 18, 14, 4, 26 | 6, 5, 2, 0, 8, 24, 10, 3, 25, 4, 14, 9, 18, 28, 20, 26, 17, 23 |  |  | 6, 16, 5, 7, 2, 8, 0, 3, 4, 12, 79, 81, 13, 17, 10, 25 | 31, 9, 30, 82, 70, 38, 81, 53, 23, 1, 6, 18, 61, 17, 5, 15, 32, 19, 54, 39 | 5, 6, 31, 80, 34, 70, 30, 79, 73, 23, 81 |
| KNN-features | 27, 12, 23, 29, 28, 5 | 29, 12, 28, 11, 1, 17, 13, 23, 25, 5, 6, 9, 7, 19, 21, 27 | 6, 5, 2, 0, 8, 24, 10, 3, 25, 4, 14, 9, 18, 28, 20, 26, 17, 23 |  |  | 6, 16, 5, 7, 2, 8, 0, 3, 4, 12, 79, 81, 13, 17, 10, 25, 70 | 31, 9, 30, 82, 70, 38, 81, 53, 23, 1, 6, 18, 61, 17, 5, 15 | 5, 6, 31, 80, 34, 70, 30, 79, 73, 23, 81 |
| Naive Bayes-features | 6, 5, 27, 26, 11 | 29, 12, 28, 11, 1, 17, 13, 23, 25, 5, 6 | 6, 5, 2, 0, 8, 24, 10, 3, 25, 4, 14, 9, 18, 28, 20, 26, 17, 23, 13, 22, 29 |  |  | 6, 16, 5, 7, 2, 8, 0, 3, 4, 12, 79, 81, 13, 17, 10, 25, 70 | 31, 9, 30, 82, 70, 38, 81, 53, 23, 1, 6, 18, 61, 17, 5, 15, 32 | 5, 6, 31, 80, 34, 70, 30, 79, 73, 23, 81, 15, 64, 60 |
| Adaboost-features | 6, 5, 7, 11, 26, 24, 19, 25, 15 | 29, 12, 28, 11, 1, 17, 13, 23, 25, 5 | 6, 5, 2 |  |  | 6, 16, 5, 7 | 31, 9, 30, 82, 70, 38 | 70, 80, 31, 5, 81, 15 |
| Gradient Boosting-features | 6, 5, 22, 26, 11, 27, 24, 7, 19, 12, 23, 0, 9, 29, 28, 2, 21 | 29, 12, 28, 11, 1, 17, 13, 23, 25, 5, 6, 9, 7, 19, 21, 27, 2, 18, 14 | 6, 5, 2, 0 |  |  | 6, 16, 5, 7, 2 | 31, 9, 30, 82, 70 | 70, 80, 31, 5, 81, 15 |
| MLP-features | 27, 5, 12, 23, 28, 26, 6 | 29, 12, 28, 11, 1, 17, 13, 23, 25, 5, 6, 9, 7, 19 | 6, 5, 2, 0, 8, 24, 10, 3, 25, 4, 14, 9, 18, 28, 20, 26, 17, 23, 13, 22 |  |  | 6, 16, 5, 7, 2, 8, 0, 3, 4, 12, 79, 81, 13, 17, 10, 25, 70, 40 | 31, 9, 30, 82, 70, 38, 81, 53, 23, 1, 6, 18, 61, 17, 5, 15, 32, 19, 54, 39 | 5, 6, 31, 80, 34, 70, 30, 79, 73, 23, 81, 15 |
| Decision Tree-alpha | 0.009686207 |  |  |  |  |  |  | 0.011151724 |
| Random Forest-alpha | 0.004037931 |  |  |  |  |  |  | 0.004665517 |
| SVM-alpha | 0.001 |  |  |  |  |  |  | 0.004317241 |
| KNN-alpha | 0.009372414 |  |  |  |  |  |  | 0.004317241 |
| Naive Bayes-alpha | 0.003724138 |  |  |  |  |  |  | 0.004317241 |
| Adaboost-alpha | 0.000696667 |  |  |  |  |  |  | 0.007734483 |
| Gradient Boosting-alpha | 0.002468966 |  |  |  |  |  |  | 0.007734483 |
| MLP-alpha | 0.006234483 |  |  |  |  |  |  | 0.004317241 |
| Decision Tree-components |  |  |  | 1 | 4 |  |  |  |
| Random Forest-components |  |  |  | 25 | 16 |  |  |  |
| SVM-components |  |  |  | 12 | 4 |  |  |  |
| KNN-components |  |  |  | 10 | 5 |  |  |  |
| Naive Bayes-components |  |  |  | 17 | 24 |  |  |  |
| Adaboost-components |  |  |  | 19 | 19 |  |  |  |
| Gradient Boosting-components |  |  |  | 27 | 15 |  |  |  |
| MLP-components |  |  |  | 7 | 10 |  |  |  |

**Supplementary Material S9: SHAP Summary Plots**

Decision Tree:

Random Forest:

Support Vector Machine:

K Nearest Neighbour:

Naive Bayes:

Adaboost:

Gradient Boosting:

Multi-layer Perceptron:
