# Supplementary material for: Associations between training behaviors and injuries in performance-oriented parkrunners using traditional statistical methods and machine learning
Source: PLoS One. 2026 Aug 19;21(8):e0350075. doi: 10.1371/journal.pone.0350075 (PMC13489425; doi:10.1371/journal.pone.0350075)
Supplement: Supplementary file 2 [file pone.0350075.s003.docx]

Editor comments:

1.Please ensure that your manuscript meets PLOS ONE's style requirements, including those for file naming. The PLOS ONE style templates can be found at

<https://journals.plos.org/plosone/s/file?id=wjVg/PLOSOne_formatting_sample_main_body.pdf> and

[https://journals.plos.org/plosone/s/file?id=ba62/PLOSOne_formatting_sample_title_authors_affiliations.pdf](https://track.editorialmanager.com/CL0/https:%2F%2Fjournals.plos.org%2Fplosone%2Fs%2Ffile%3Fid=ba62%2FPLOSOne_formatting_sample_title_authors_affiliations.pdf/1/010f019f01d35819-2acf48ed-d7e3-4923-86d1-a05840f6af48-000000/J6GQqdod3r6PU4TSSd9MvSS7kFHRbz59mcF2S5TIDpw=258)

These requirements have now been applied to the manuscript.

2. The ethical approval number(s) listed in the manuscript and/or submission metadata does not match the approval number on the ethical approval document you provided. Please ensure that all approval numbers are correct.

The Institutional Ethics Sub-Committee approval number has been added to the manuscript. The parkrun Research Board did not provided an application or approval number but are not an ethics committee.

3. In the online submission form, you indicated that your data is available only on request from a third party. Please note that your Data Availability Statement is currently missing [the name of the third party contact or institution / contact details for the third party, such as an email address or a link to where data requests can be made]. Please update your statement with the missing information.

The data availability statement has now been updated with this information.

4. Please include your full ethics statement in the ‘Methods’ section of your manuscript file. In your statement, please include the full name of the IRB or ethics committee who approved or waived your study, as well as whether or not you obtained informed written or verbal consent. If consent was waived for your study, please include this information in your statement as well.

This information is all now included in section 2.1 – the first section of the Methods.

5. Please include captions for your Supporting Information files at the end of your manuscript, and update any in-text citations to match accordingly. Please see our Supporting Information guidelines for more information: [http://journals.plos.org/plosone/s/supporting-information](http://track.editorialmanager.com/CL0/http:%2F%2Fjournals.plos.org%2Fplosone%2Fs%2Fsupporting-information/1/010f019f01d35819-2acf48ed-d7e3-4923-86d1-a05840f6af48-000000/fIk9SkRhu4P8HWNx3TqsKD0e9rAeznbJv4dXeiQaHC4=258).

We have included this information at the end of our manuscript.

6. If the reviewer comments include a recommendation to cite specific previously published works, please review and evaluate these publications to determine whether they are relevant and should be cited. There is no requirement to cite these works unless the editor has indicated otherwise.

None of the reviewers recommended citing specific works.

7. Please review your reference list to ensure that it is complete and correct. If you have cited papers that have been retracted, please include the rationale for doing so in the manuscript text, or remove these references and replace them with relevant current references. Any changes to the reference list should be mentioned in the rebuttal letter that accompanies your revised manuscript. If you need to cite a retracted article, indicate the article’s retracted status in the References list and also include a citation and full reference for the retraction notice.

The reference list is complete and correct. No cited papers have been retracted.

Reviewer 1 comments:

GENERAL COMMENTS
The study addresses an important topic, the notion that strength and conditioning activities including stretching and resistance training can prevent injury. The focus on Park Run participants is justified by the large participation levels in this activity. The paper is generally well written and structured and well designed. The use of Machine Learning algorithms adds an element of novelty to the paper but as I am not expert in this area, I cannot really comment with any authority on this aspect of the paper. Overall I have no major concerns about the paper and I think it adds to the literature on the topic.

Thank you very much for your acknowledgement and your helpful suggestions, which we believe have critically pointed out this manuscript’s limitations.

I have however one overall minor concern that the literature on this topic is dominated by a reliance on cross sectional correlation based studies which rely associations of S&C training with reduced injury incidence rather than more robust designs such and prospective randomized clinical trial based designs. My concern is that this study in it current form (especially the discussion) does does not do enough to address this or acknowledge this limitation.

We fully agree that the design of this study is a limitation and not as strong as prospectively designed studies. We have added wording to the limitations section to emphasize this issue alongside the current wording that recognizes the drawback of the study design. A statement has also been added to the ‘perspective’ paragraph to ensure this point is made in concluding remarks.

SPECIFIC COMMENTS
ln 116: Has ML really predicted injury or have these predictions been coincidence? Does this stand up against prospective trial evidence? perhaps some critical evaluation is required?

Thank you for raising this point. We have added some critical evaluations covering the current lack of externally validated ML models in the field, which raises questions regarding whether the predictions were merely coincidental and/or due to overfitting.

Ln 304-306: for clarity here: I assume this implies that lower injury incidence increased with increases in S&C and Yoga? (A matter for the discussion :Is this consistent with other studies?)

Yes, it implies that injury incidence was higher for runners who regularly conducted strength training and yoga compared to those who did not regularly conduct strength training and yoga. Thank you for giving helpful advice regarding the discussion section. We have made changes to the discussion section based on your comment here and other specific suggestions below.

ln 306-308: Might this imply that S&C had no effect on participants who had no history of injury but it was associated with increases in injury prevalence on the whole sample. (implying that participants who had previous injury and did more S&C, had increased injury incidence?) Is this consistent with existing literature? This finding does not seem to be consistent with the narrative that exists amongst runners, trainers and therapists which "believes" that S&C prevents injury. This is despite the lack of prospective randomized control trial data.

We agree with your assessment. We believe the most important reason for this observation is that runners who were injured pick up S&C due to physiotherapist/coach advice and/or their own perception that S&C can help rehabilitate from and/or prevent future injuries. As per your comments below for the discussion section, we have added comparisons against existing evidence and discussed what we believe these results indicate (lines 449-453).

DISCUSSION
The results are well discussed and the results demonstrate little evidence that S&C and Yoga are associated with reduced incidence of injury. This appears to be a important finding because the predominant narrative is that S&C prevents injuries.
This narrative prevails despite the lack of robust evidence (Prospective trials). However, I think some additions to the discussion are needed to put this in context. Perhaps emphasize that the findings of this study contrast with existing literature, but add the limitations of existing literature relying on correlation based findings. I think this needs to be addressed. Similarly, this may be a limitation of this study as well and you may consider adding this to the discussion?

Thank you for offering these helpful comments. We agree that the currently predominant narrative that S&C can prevent RRIs lacks sufficient evidential support from the research community. We also agree that the discussion section would benefit from more comparisons with previous literature and from a deeper fixation on how the retrospective nature of this study could introduce unwanted confounders. We have now added in lines (449-453) comparisons against previous literature showing that prospective intervention trials did not find S&C to positively associate with RRIs, while a retrospective survey study similar to ours found stretching before running to positively correlate with RRIs. We also added within the limitations section in line (511-514) to stress that the positive associations between S&C and RRIs were likely due to reverse causality.

Reviewer 2 comments:

The work is in the manuscript is interesting and worthwhile. The scope of the study is based on parkrun participants which is an extensive international grouping. I have a number of small queries and comments which are mainly looking for clarifications or correcting typos. These are listed below and the page numbers refer to the pdf page and give the line number.

Thank you very much for your positive comments, which we really appreciate. We find your comments below helpful and constructive and believe the amendments/additions we have made as a result have resulted in an improved manuscript.

The work could impact the approach to training that participants plan to take when training for parkrun or similar length runs/races.

We fully agree. Thanks again for your acknowledgement.

Abstract. I would give the p-value after the phi value in "Resistance training (p=0.018, Φ=0.099) and stretching/yoga (p=0.023, Φ=0.095) were positively associated with RRIs."

Thank you for your suggestion. We agree that p values should better appear after phi values. We have corrected these within the abstract as well as the results sections.

Page 10. Line 115 "the application of ML to training and injury data in endurance sports is scarce.[15]" Is parkrun considered to be an endurance sport? Would "individual sports" be a better phrase?

Thank you for pointing this out. parkrun is 5 km and is indeed borderline in terms of being categorized as “endurance sport”. We have changed the expression to “individual sports” as suggested.

Page 13. Line 175. I think saying a little bit more about phi is useful for a more casual reader.

Many thanks for the advice. We have added a brief explanation of the phi coefficient within the manuscript text (lines 181-182).

Page 14. Line 2017. Principal not principle

Thank you for noticing this. Sorry about the typo; it is now corrected.

Page 14. Section 2.4.2. There are a number of implementations of the methods in Python. It would be worth being more explicit about which functions/libraries were used.

Thank you for raising this point. We have added at the beginning of the section that ML-related implementations were conducted using the sklearn module in python (lines 225-226).

Page 14. Line 223. What is n=27 referring to?

27 refers to the number of components that account for 90% total energy (explanatory power) within the PCA. Sorry for the lack of clarity in our expression; we have adjusted our expression within the manuscript text to be more explicit regarding what n=27 refers to.

Page 15. Line 227. Should "Relief" be "random forests"?

We are sorry about the confusion here, “decision tree and Relief” here refer to feature selection methods instead of algorithms used to construct the classifier. We have added descriptions in-text to make this more explicit. The overall implementation was that we used feature selection methods to rank features, and then tested features incrementally (top 1, top 1+2, etc.) using stratified 10-fold cross-validation based on the ranking for each classifier. Decision tree was employed both as a feature selection method and as a classifier.

Page 20. Section 3.5. This section is very hard to follow. It is listing what was done in a somewhat haphazard way. A more careful description is needed. LASSO on what? LASSO is a linear method but the others are non-linear

We are sorry about the confusion. We have added a more detailed explanation here about the feature selection process. LASSO was used to rank all features, and then the features were tested incrementally on all classifiers, yielding the feature subset with the highest AUC during stratified 10-fold cross-validation. It turned out that the maximum AUC-achieving feature subset appeared in LASSO-ranked feature subsets instead of other ranking methods (decision tree, Relief, PCA) for all classifiers.
