# Supplementary material for: Associations between training behaviors and injuries in performance-oriented parkrunners using traditional statistical methods and machine learning
Source: PLoS One. 2026 Aug 19;21(8):e0350075. doi: 10.1371/journal.pone.0350075 (PMC13489425; doi:10.1371/journal.pone.0350075)
Supplement: Peer review history

# Peer review: Decision Letter 0

**DOI:** 10.1371/journal.pone.0350075.r001

**Authors:** Laura-Anne Furlong

25 Jun 2026

Dear Dr. Blagrove,

Thank you for submitting your manuscript to PLOS ONE. After careful consideration, we feel that it has merit but does not fully meet PLOS ONE’s publication criteria as it currently stands. Therefore, we invite you to submit a revised version of the manuscript that addresses the points raised during the review process.

Please submit your revised manuscript by Jul 25 2026 11:59PM. If you will need more time than this to complete your revisions, please reply to this message or contact the journal office at plosone@plos.org. When you're ready to submit your revision, log on to https://www.editorialmanager.com/pone/ and select the 'Submissions Needing Revision' folder to locate your manuscript file.

- A letter that responds to each point raised by the academic editor and reviewer(s). You should upload this letter as a separate file labeled 'Response to Reviewers'.
- A marked-up copy of your manuscript that highlights changes made to the original version. You should upload this as a separate file labeled 'Revised Manuscript with Track Changes'.
- An unmarked version of your revised paper without tracked changes. You should upload this as a separate file labeled 'Manuscript'.

If you would like to make changes to your financial disclosure, please include your updated statement in your cover letter. Guidelines for resubmitting your figure files are available below the reviewer comments at the end of this letter.

If applicable, we recommend that you deposit your laboratory protocols in protocols.io to enhance the reproducibility of your results. Protocols.io assigns your protocol its own identifier (DOI) so that it can be cited independently in the future. For instructions see: https://journals.plos.org/plosone/s/submission-guidelines#loc-laboratory-protocols. Additionally, PLOS ONE offers an option for publishing peer-reviewed Lab Protocol articles, which describe protocols hosted on protocols.io. Read more information on sharing protocols at https://plos.org/protocols?utm_medium=editorial-email&utm_source=authorletters&utm_campaign=protocols.

As the corresponding author, your ORCID iD is verified in the submission system and will appear in the published article. PLOS supports the use of ORCID, and we encourage all coauthors to register for an ORCID iD and use it as well. Please encourage your coauthors to verify their ORCID iD within the submission system before final acceptance, as unverified ORCID iDs will not appear in the published article. *Only*  the individual author can complete the verification step; PLOS staff *cannot*  verify ORCID iDs on behalf of authors.

We look forward to receiving your revised manuscript.

Kind regards,

Laura-Anne Marie Furlong

Academic Editor

PLOS One

Journal Requirements:

When submitting your revision, we need you to address these additional requirements.

1.Please ensure that your manuscript meets PLOS ONE's style requirements, including those for file naming. The PLOS ONE style templates can be found at

https://journals.plos.org/plosone/s/file?id=wjVg/PLOSOne_formatting_sample_main_body.pdf and

https://journals.plos.org/plosone/s/file?id=ba62/PLOSOne_formatting_sample_title_authors_affiliations.pdf

2. The ethical approval number(s) listed in the manuscript and/or submission metadata does not match the approval number on the ethical approval document you provided. Please ensure that all approval numbers are correct.

3. In the online submission form, you indicated that your data is available only on request from a third party. Please note that your Data Availability Statement is currently missing [the name of the third party contact or institution / contact details for the third party, such as an email address or a link to where data requests can be made]. Please update your statement with the missing information.

4. Please include your full ethics statement in the ‘Methods’ section of your manuscript file. In your statement, please include the full name of the IRB or ethics committee who approved or waived your study, as well as whether or not you obtained informed written or verbal consent. If consent was waived for your study, please include this information in your statement as well.

5. Please include captions for your Supporting Information files at the end of your manuscript, and update any in-text citations to match accordingly. Please see our Supporting Information guidelines for more information: http://journals.plos.org/plosone/s/supporting-information.

6. If the reviewer comments include a recommendation to cite specific previously published works, please review and evaluate these publications to determine whether they are relevant and should be cited. There is no requirement to cite these works unless the editor has indicated otherwise.

7. Please review your reference list to ensure that it is complete and correct. If you have cited papers that have been retracted, please include the rationale for doing so in the manuscript text, or remove these references and replace them with relevant current references. Any changes to the reference list should be mentioned in the rebuttal letter that accompanies your revised manuscript. If you need to cite a retracted article, indicate the article’s retracted status in the References list and also include a citation and full reference for the retraction notice.

[Note: HTML markup is below. Please do not edit.]

Reviewer's Responses to Questions

**Comments to the Author**

1. Is the manuscript technically sound, and do the data support the conclusions?

Reviewer #1: Yes

Reviewer #2: Yes

**********

2. Has the statistical analysis been performed appropriately and rigorously?

Reviewer #1: I Don't Know

Reviewer #2: Yes

**********

3. Have the authors made all data underlying the findings in their manuscript fully available?

The PLOS Data policy

Reviewer #1: Yes

Reviewer #2: Yes

**********

4. Is the manuscript presented in an intelligible fashion and written in standard English?

Reviewer #1: Yes

Reviewer #2: Yes

**********

Reviewer #1: GENERAL COMMENTS

The study addresses an important topic, the notion that strength and conditioning activities including stretching and resistance training can prevent injury. The focus on Park Run participants is justified by the large participation levels in this activity. The paper is generally well written and structured and well designed. The use of Machine Learning algorithms adds an element of novelty to the paper but as I am not expert in this area, I cannot really comment with any authority on this aspect of the paper. Overall I have no major concerns about the paper and I think it adds to the literature on the topic. I have however one overall minor concern that the literature on this topic is dominated by a reliance on cross sectional correlation based studies which rely associations of S&C training with reduced injury incidence rather than more robust designs such and prospective randomized clinical trial based designs. My concern is that this study in it current form (especially the discussion) does does not do enough to address this or acknowledge this limitation.

SPECIFIC COMMENTS

ln 116: Has ML really predicted injury or have these predictions been coincidence? Does this stand up against prospective trial evidence? perhaps some critical evaluation is required?

Ln 304-306: for clarity here: I assume this implies that lower injury incidence increased with increases in S&C and Yoga? (A matter for the discussion :Is this consistent with other studies?)

ln 306-308: Might this imply that S&C had no effect on participants who had no history of injury but it was associated with increases in injury prevalence on the whole sample. (implying that participants who had previous injury and did more S&C, had increased injury incidence?) Is this consistent with existing literature? This finding does not seem to be consistent with the narrative that exists amongst runners, trainers and therapists which "believes" that S&C prevents injury. This is despite the lack of prospective randomized control trial data.

DISCUSSION

The results are well discussed and the results demonstrate little evidence that S&C and Yoga are associated with reduced incidence of injury. This appears to be a important finding because the predominant narrative is that S&C prevents injuries.

This narrative prevails despite the lack of robust evidence (Prospective trials). However, I think some additions to the discussion are needed to put this in context. Perhaps emphasize that the findings of this study contrast with existing literature, but add the limitations of existing literature relying on correlation based findings. I think this needs to be addressed. Similarly, this may be a limitation of this study as well and you may consider adding this to the discussion?

Reviewer #2: The work is in the manuscript is interesting and worthwhile. The scope of the study is based on parkrun participants which is an extensive international grouping. I have a number of small queries and comments which are mainly looking for clarifications or correcting typos. These are listed below and the page numbers refer to the pdf page and give the line number.

The work could impact the approach to training that participants plan to take when training for parkrun or similar length runs/races.

Abstract. I would give the p-value after the phi value in "Resistance training (p=0.018, Φ=0.099) and stretching/yoga (p=0.023, Φ=0.095) were positively associated with RRIs."

Page 10. Line 115 "the application of ML to training and injury data in endurance sports is scarce.[15]" Is parkrun considered to be an endurance sport? Would "individual sports" be a better phrase?

Pagge 13. Line 175. I think saying a little bit more about phi is useful for a more casual reader.

Page 14. Line 2017. Principal not principle

Page 14. Section 2.4.2. There are a number of implementations of the methods in Python. It would be worth being more explicit about which functions/libraries were used.

Page 14. Line 223. What is n=27 referring to?

Page 15. Line 227. Should "Relief" be "random forests"?

Page 20. Section 3.5. This section is very hard to follow. It is listing what was done in a somewhat haphazard way. A more careful description is needed. LASSO on what? LASSO is a linear method but the others are non-linear

**********

what does this mean?). If published, this will include your full peer review and any attached files.

If you choose “no”, your identity will remain anonymous but your review may still be made public.

**Do you want your identity to be public for this peer review?**  For information about this choice, including consent withdrawal, please see our Privacy Policy

Reviewer #1: No

Reviewer #2: No

**********

[NOTE: If reviewer comments were submitted as an attachment file, they will be attached to this email and accessible via the submission site. Please log into your account, locate the manuscript record, and check for the action link "View Attachments". If this link does not appear, there are no attachment files.]

To ensure your figures meet our technical requirements, please review our figure guidelines: https://journals.plos.org/plosone/s/figures

You may also use PLOS’s free figure tool, NAAS, to help you prepare publication quality figures: https://journals.plos.org/plosone/s/figures#loc-tools-for-figure-preparation.

NAAS will assess whether your figures meet our technical requirements by comparing each figure against our figure specifications.

# Author comment: Author response to Decision Letter 1

**DOI:** 10.1371/journal.pone.0350075.r002

24 Jul 2026

Editor comments:

1.Please ensure that your manuscript meets PLOS ONE's style requirements, including those for file naming. The PLOS ONE style templates can be found at

https://journals.plos.org/plosone/s/file?id=wjVg/PLOSOne_formatting_sample_main_body.pdf and

https://journals.plos.org/plosone/s/file?id=ba62/PLOSOne_formatting_sample_title_authors_affiliations.pdf

Authors: These requirements have now been applied to the manuscript.

2. The ethical approval number(s) listed in the manuscript and/or submission metadata does not match the approval number on the ethical approval document you provided. Please ensure that all approval numbers are correct.

Authors: The Institutional Ethics Sub-Committee approval number has been added to the manuscript. The parkrun Research Board did not provided an application or approval number but are not an ethics committee.

3. In the online submission form, you indicated that your data is available only on request from a third party. Please note that your Data Availability Statement is currently missing [the name of the third party contact or institution / contact details for the third party, such as an email address or a link to where data requests can be made]. Please update your statement with the missing information.

Authors: The data availability statement has now been updated with this information.

4. Please include your full ethics statement in the ‘Methods’ section of your manuscript file. In your statement, please include the full name of the IRB or ethics committee who approved or waived your study, as well as whether or not you obtained informed written or verbal consent. If consent was waived for your study, please include this information in your statement as well.

Authors: This information is all now included in section 2.1 – the first section of the Methods.

5. Please include captions for your Supporting Information files at the end of your manuscript, and update any in-text citations to match accordingly. Please see our Supporting Information guidelines for more information: http://journals.plos.org/plosone/s/supporting-information.

Authors: We have included this information at the end of our manuscript.

6. If the reviewer comments include a recommendation to cite specific previously published works, please review and evaluate these publications to determine whether they are relevant and should be cited. There is no requirement to cite these works unless the editor has indicated otherwise.

Authors: None of the reviewers recommended citing specific works.

7. Please review your reference list to ensure that it is complete and correct. If you have cited papers that have been retracted, please include the rationale for doing so in the manuscript text, or remove these references and replace them with relevant current references. Any changes to the reference list should be mentioned in the rebuttal letter that accompanies your revised manuscript. If you need to cite a retracted article, indicate the article’s retracted status in the References list and also include a citation and full reference for the retraction notice.

Authors: The reference list is complete and correct. No cited papers have been retracted.

Reviewer 1 comments:

GENERAL COMMENTS

The study addresses an important topic, the notion that strength and conditioning activities including stretching and resistance training can prevent injury. The focus on Park Run participants is justified by the large participation levels in this activity. The paper is generally well written and structured and well designed. The use of Machine Learning algorithms adds an element of novelty to the paper but as I am not expert in this area, I cannot really comment with any authority on this aspect of the paper. Overall I have no major concerns about the paper and I think it adds to the literature on the topic.

Authors: Thank you very much for your acknowledgement and your helpful suggestions, which we believe have critically pointed out this manuscript’s limitations.

I have however one overall minor concern that the literature on this topic is dominated by a reliance on cross sectional correlation based studies which rely associations of S&C training with reduced injury incidence rather than more robust designs such and prospective randomized clinical trial based designs. My concern is that this study in it current form (especially the discussion) does does not do enough to address this or acknowledge this limitation.

Authors: We fully agree that the design of this study is a limitation and not as strong as prospectively designed studies. We have added wording to the limitations section to emphasize this issue alongside the current wording that recognizes the drawback of the study design. A statement has also been added to the ‘perspective’ paragraph to ensure this point is made in concluding remarks.

SPECIFIC COMMENTS

ln 116: Has ML really predicted injury or have these predictions been coincidence? Does this stand up against prospective trial evidence? perhaps some critical evaluation is required?

Authors: Thank you for raising this point. We have added some critical evaluations covering the current lack of externally validated ML models in the field, which raises questions regarding whether the predictions were merely coincidental and/or due to overfitting.

Ln 304-306: for clarity here: I assume this implies that lower injury incidence increased with increases in S&C and Yoga? (A matter for the discussion :Is this consistent with other studies?)

Authors: Yes, it implies that injury incidence was higher for runners who regularly conducted strength training and yoga compared to those who did not regularly conduct strength training and yoga. Thank you for giving helpful advice regarding the discussion section. We have made changes to the discussion section based on your comment here and other specific suggestions below.

ln 306-308: Might this imply that S&C had no effect on participants who had no history of injury but it was associated with increases in injury prevalence on the whole sample. (implying that participants who had previous injury and did more S&C, had increased injury incidence?) Is this consistent with existing literature? This finding does not seem to be consistent with the narrative that exists amongst runners, trainers and therapists which "believes" that S&C prevents injury. This is despite the lack of prospective randomized control trial data.

Authors: We agree with your assessment. We believe the most important reason for this observation is that runners who were injured pick up S&C due to physiotherapist/coach advice and/or their own perception that S&C can help rehabilitate from and/or prevent future injuries. As per your comments below for the discussion section, we have added comparisons against existing evidence and discussed what we believe these results indicate (lines 449-453).

DISCUSSION

The results are well discussed and the results demonstrate little evidence that S&C and Yoga are associated with reduced incidence of injury. This appears to be a important finding because the predominant narrative is that S&C prevents injuries.This narrative prevails despite the lack of robust evidence (Prospective trials). However, I think some additions to the discussion are needed to put this in context. Perhaps emphasize that the findings of this study contrast with existing literature, but add the limitations of existing literature relying on correlation based findings. I think this needs to be addressed. Similarly, this may be a limitation of this study as well and you may consider adding this to the discussion?

Authors: Thank you for offering these helpful comments. We agree that the currently predominant narrative that S&C can prevent RRIs lacks sufficient evidential support from the research community. We also agree that the discussion section would benefit from more comparisons with previous literature and from a deeper fixation on how the retrospective nature of this study could introduce unwanted confounders. We have now added in lines (449-453) comparisons against previous literature showing that prospective intervention trials did not find S&C to positively associate with RRIs, while a retrospective survey study similar to ours found stretching before running to positively correlate with RRIs. We also added within the limitations section in line 511-514 to stress that the positive associations between S&C and RRIs were likely due to reverse causality.

Reviewer 2 comments:

The work is in the manuscript is interesting and worthwhile. The scope of the study is based on parkrun participants which is an extensive international grouping. I have a number of small queries and comments which are mainly looking for clarifications or correcting typos. These are listed below and the page numbers refer to the pdf page and give the line number.

Authors: Thank you very much for your positive comments, which we really appreciate. We find your comments below helpful and constructive and believe the amendments/additions we have made as a result have resulted in an improved manuscript.

The work could impact the approach to training that participants plan to take when training for parkrun or similar length runs/races.

Authors: We fully agree. Thanks again for your acknowledgement.

Abstract. I would give the p-value after the phi value in "Resistance training (p=0.018, Φ=0.099) and stretching/yoga (p=0.023, Φ=0.095) were positively associated with RRIs."

Authors: Thank you for your suggestion. We agree that p values should better appear after phi values. We have corrected these within the abstract as well as the results sections.

Page 10. Line 115 "the application of ML to training and injury data in endurance sports is scarce.[15]" Is parkrun considered to be an endurance sport? Would "individual sports" be a better phrase?

Authors: Thank you for pointing this out. parkrun is 5 km and is indeed borderline in terms of being categorized as “endurance sport”. We have changed the expression to “individual sports” as suggested.

Page 13. Line 175. I think saying a little bit more about phi is useful for a more casual reader.

Authors: Many thanks for the advice. We have added a brief explanation of the phi coefficient within the manuscript text (lines 181-182).

Page 14. Line 2017. Principal not principle

Authors: Thank you for noticing this. Sorry about the typo; it is now corrected.

Page 14. Section 2.4.2. There are a number of implementations of the methods in Python. It would be worth being more explicit about which functions/libraries were used.

Authors: Thank you for raising this point. We have added at the beginning of the section that ML-related implementations were conducted using the sklearn module in python (lines 225-226).

Page 14. Line 223. What is n=27 referring to?

Authors: 27 refers to the number of components that account for 90% total energy (explanatory power) within the PCA. Sorry for the lack of clarity in our expression; we have adjusted our expression within the manuscript text to be more explicit regarding what n=27 refers to.

Page 15. Line 227. Should "Relief" be "random forests"?

Authors: We are sorry about the confusion here, “decision tree and Relief” here refer to feature selection methods instead of algorithms used to construct the classifier. We have added descriptions in-text to make this more explicit. The overall implementation was that we used feature selection methods to rank features, and then tested features incrementally (top 1, top 1+2, etc.) using stratified 10-fold cross-validation based on the ranking for each classifier. Decision tree was employed both as a feature selection method and as a classifier.

Page 20. Section 3.5. This section is very hard to follow. It is listing what was done in a somewhat haphazard way. A more careful description is needed. LASSO on what? LASSO is a linear method but the others are non-linear

Authors: We are sorry about the confusion. We have added a more detailed explanation here about the feature selection process. LASSO was used to rank all features, and then the features were tested incrementally on all classifiers, yielding the feature subset with the highest AUC during stratified 10-fold cross-validation. It turned out that the maximum AUC-achieving feature subset appeared in LASSO-ranked feature subsets instead of other ranking methods (decision tree, Relief, PCA) for all classifiers.

Attachment Submitted filename: response to reviewer comments.docx

# Peer review: Decision Letter 1

**DOI:** 10.1371/journal.pone.0350075.r003

**Authors:** Laura-Anne Furlong

5 Aug 2026

Associations between training behaviors and injuries in performance-oriented parkrunners using traditional statistical methods and machine learning

PONE-D-26-21232R1

Dear Dr. Blagrove,

We’re pleased to inform you that your manuscript has been judged scientifically suitable for publication and will be formally accepted for publication once it meets all outstanding technical requirements.

Within one week, you’ll receive an e-mail detailing the required amendments. When these have been addressed, you’ll receive a formal acceptance letter and your manuscript will be scheduled for publication.

An invoice will be generated when your article is formally accepted. Please note, if your institution has a publishing partnership with PLOS and your article meets the relevant criteria, all or part of your publication costs will be covered. Please make sure your user information is up-to-date by logging into Editorial Manager at Editorial Manager® and clicking the ‘Update My Information' link at the top of the page. For questions related to billing, please contact billing support.

If your institution or institutions have a press office, please notify them about your upcoming paper to help maximize its impact. If they’ll be preparing press materials, please inform our press team as soon as possible -- no later than 48 hours after receiving the formal acceptance. Your manuscript will remain under strict press embargo until 2 pm Eastern Time on the date of publication. For more information, please contact onepress@plos.org.

Kind regards,

Laura-Anne Marie Furlong

Academic Editor

PLOS One

Additional Editor Comments (optional):

Reviewers' comments:

Reviewer's Responses to Questions

**Comments to the Author**

Reviewer #1: All comments have been addressed

Reviewer #2: All comments have been addressed

**********

2. Is the manuscript technically sound, and do the data support the conclusions?

Reviewer #1: Yes

Reviewer #2: Yes

**********

3. Has the statistical analysis been performed appropriately and rigorously?

Reviewer #1: Yes

Reviewer #2: Yes

**********

4. Have the authors made all data underlying the findings in their manuscript fully available?

The PLOS Data policy

Reviewer #1: Yes

Reviewer #2: Yes

**********

5. Is the manuscript presented in an intelligible fashion and written in standard English?

Reviewer #1: Yes

Reviewer #2: Yes

**********

Reviewer #1: (No Response)

Reviewer #2: Thank you for addressing the points raised. I am happy with the revised manuscript. Thank you for your effort.

**********

what does this mean?). If published, this will include your full peer review and any attached files.

If you choose “no”, your identity will remain anonymous but your review may still be made public.

**Do you want your identity to be public for this peer review?**  For information about this choice, including consent withdrawal, please see our Privacy Policy

Reviewer #1: **Yes:**  Andrew J Harrison

Reviewer #2: No

**********

# Editor report: Acceptance letter

**DOI:** 10.1371/journal.pone.0350075.r004

**Authors:** Laura-Anne Furlong

PONE-D-26-21232R1

PLOS One

Dear Dr. Blagrove,

I'm pleased to inform you that your manuscript has been deemed suitable for publication in PLOS One. Congratulations! Your manuscript is now being handed over to our production team.

At this stage, our production department will prepare your paper for publication. This includes ensuring the following:

* All references, tables, and figures are properly cited

* All relevant supporting information is included in the manuscript submission,

* There are no issues that prevent the paper from being properly typeset

You will receive further instructions from the production team, including instructions on how to review your proof when it is ready. Please keep in mind that we are working through a large volume of accepted articles, so please give us a few days to review your paper and let you know the next and final steps.

Lastly, if your institution or institutions have a press office, please let them know about your upcoming paper now to help maximize its impact. If they'll be preparing press materials, please inform our press team within the next 48 hours. Your manuscript will remain under strict press embargo until 2 pm Eastern Time on the date of publication. For more information, please contact onepress@plos.org.

You will receive an invoice from PLOS for your publication fee after your manuscript has reached the completed accept phase. If you receive an email requesting payment before acceptance or for any other service, this may be a phishing scheme. Learn how to identify phishing emails and protect your accounts at https://explore.plos.org/phishing.

If we can help with anything else, please email us at customercare@plos.org.

Thank you for submitting your work to PLOS ONE and supporting open access.

Kind regards,

PLOS ONE Editorial Office Staff

on behalf of

Dr. Laura-Anne Marie Furlong

Academic Editor

PLOS One
